# A comparative analysis of the adaptability of salt stress between two flax (*Linum usitatissimum* L.) genotypes, Flanders and Astella, having contrasting lignan contents

**DOI:** 10.1007/s00425-025-04861-4

**Published:** 2025-11-10

**Authors:** Anirban Jyoti Debnath, Ľubomír Harenčár, Matúš Kučka, Marek Kovár, Eva Ivanišová, Veronika Mistríková, Ján Gažo, Katarína Ražná

**Affiliations:** 1 Institute of Plant and Environmental Sciences, Faculty of Agrobiology and Food Resources, Slovak University of Agriculture in Nitra, Trieda Andreja Hlinku 2, 949 76 Nitra, Slovakia; 2 Institute of Food Sciences, Faculty of Biotechnology and Food Sciences, Slovak University of Agriculture in Nitra, Trieda Andreja Hlinku 2, 949 76 Nitra, Slovakia; 3 Institute of Plant Genetics and Biotechnology, Plant Science and Biodiversity Centre, Slovak Academy of Sciences, P. O. Box 39 A, Akademická 2, 950 07 Nitra, Slovakia; 4 Department of Genetics, Institute of Biology and Ecology, Faculty of Science, Pavol Jozef Šafárik University, Mánesova 23, 041 54 Košice, Slovakia

**Keywords:** Biochemical analysis, MicroRNA, Morphological analysis, Reactive oxygen species, Secoisolariciresinol diglucoside, Two-tailed quantitative PCR

## Abstract

**Main conclusion:**

A comparative analysis of NaCl-stressed flax genotypes reveals that microRNA828a, microRNA399g, microRNA168a, catalase, shoot length, and shoot dry weight are the most influential salt stress-responsive variables irrespective of the lignan (secoisolariciresinol diglucoside) content declared for the genotypes.

**Abstract:**

Lignans are powerful antioxidants and plant defence molecules whose roles in mitigating salt stress are rarely studied or understood, particularly in flax (*Linum usitatissimum* L.). Flax is a rich source of lignans. This study assessed the response to salt stress in two flax genotypes, Flanders and Astella. Astella has a higher content of the lignan secoisolariciresinol diglucoside (SDG) than Flanders. The 3-week-old flax plants were subjected to 100 mM NaCl salt stress for 1 week. Morphological analyses revealed that the growth of Flanders was more suppressed under stress, indicating resource-saving behaviour compared to Astella. Salt stress caused Astella to produce more reactive oxygen species (ROS) and incur more cell damage than Flanders. Flanders exhibited comparatively higher levels of antioxidants, osmoprotection machinery, and lignan-related microRNAs, suggesting its enhanced ROS scavenging and superior cellular protection capabilities than Astella. However, multivariate analysis could not provide evidence for the direct involvement of lignans in stress adaptation. Instead, it was hypothesised that microRNAs play a pleiotropic role in the adaptation to salinity. The results demonstrated Flanders’ superiority to Astella in salt stress mitigation. The findings could be used to improve the salinity tolerance of flax and other crop plants in future research.

**Supplementary Information:**

The online version contains supplementary material available at 10.1007/s00425-025-04861-4.

## Introduction

The abiotic stress factors caused by rapid climate change are leading to annual crop yield losses of 51–82% in global agriculture (Onyekachi et al. 2019; Oshunsanya et al. 2019). Salinity, a major abiotic stress, is characterised by an elevated concentration of soluble salts in soil. Soils are classified as saline when the electrical conductivity (ECe) is 4 decisiemens per metre (dS/m) or more, which is equivalent to approximately 40 mM NaCl and generates an osmotic pressure of approximately 0.2 MPa (Munns and Tester 2008). It is estimated that approximately 50% of all arable land could be affected by salinisation by 2050 (FAO 2021). This alarming situation necessitates research on salinity-stress-resilient crops (UN 2025).

A high concentration of salts in the soil restricts the ability of plants to uptake water and nutrients, thus leading to a physiological drought condition (Ma et al. 2020). Consequently, the salt solute potential of plants becomes imbalanced. To counteract the situation, plants channelise photosynthates to synthesise and accumulate osmolytes such as proline and glycine betaine, thereby balancing the salt solute potential and favouring a stable water flux (Türkan and Demiral 2009). However, diverting cellular resources away from the key physiological processes, such as photosynthesis, results in a drastic reduction in the growth rate of plants affected by salinity. Growth arrest under stress is defined as an adaptive mechanism that enables plants to preserve carbohydrates for sustained metabolism, prolonged energy supply, and better recovery after stress relief (Bartels and Sunkar 2005).

Salinity stress disrupts the cellular balance between energy intake and consumption. Consequently, electrons begin to leak from the electron transport chains of photosynthesis (chloroplasts), respiration (mitochondria), and photorespiration (peroxisomes). These leaked electrons oxidise oxygen molecules and generate a large amount of reactive oxygen species (ROS), which damage molecular and cellular components by oxidising biomolecules (lipids, carbohydrates, proteins, enzymes, and DNA), and ultimately cause plant death (Hasanuzzaman et al. 2019; Mekawy et al. 2020). To combat this fatal situation, plant cells have a well-established ROS-scavenging antioxidant defence mechanism consisting of enzymatic, non-enzymatic, and non-protein amino acid components (Gill and Tuteja 2010; Hasanuzzaman et al. 2019). The multiple hydroxyl groups of polyphenols (phenolic acids, flavonoids, stilbenoids, and lignans) also render them powerful ROS scavengers (Šamec et al. 2021).

Lignans are secondary metabolites that belong to the group of dimeric phenylpropanoids. Phenylpropanoids are phenylpropane derivatives, characterised by a structural framework compromising a propane moiety (a three-carbon chain) linked to a phenyl group. The simplest form of lignan is a phenylpropanoid dimer, in which two phenylpropane units are β-β’ linked at their carbon 8 (Teponno et al. 2016). Naturally occurring lignans have varying degrees of substitution in their phenylpropane scaffolds (Markulin et al. 2019a, b). They are present in the flowers, seeds, leaves, roots, fruits, and woody parts of higher plants, and primarily function in plant defence mechanisms (Ražná et al. 2021).

Lignans are involved in plant defence mechanisms through their antioxidant activity (Ghotbzadeh Kermani et al. 2019). The synthesis of several polyphenols, including lignans, increases under abiotic stress factors such as drought, extreme temperatures, salinity, heavy metals treatments, and UV radiation (Sharma et al. 2019; Ražná et al. 2021). Under drought stress, the lignan contents of sesame (*Sesamum indicum* L.) change in a genotype-specific manner. Moreover, the drought-tolerant sesame genotypes contain high levels of lignans, such as sesamin and/or sesamolin (Ghotbzadeh Kermani et al. 2019). Heavy metal stress-tolerant rapeseed (*Brassica napus* L.) genotypes accumulate more lignans under cadmium stress than the susceptible genotypes (Mwamba et al. 2020). Lignans also possess antibacterial, antifungal, and antifeedant properties (Zálešák et al. 2019).

MicroRNAs (miRNAs) are a class of non-coding, 19–24 nucleotide-long RNA molecules that function in the negative regulation of gene expression at the post-transcriptional level. In plants, a miRNA is loaded into the RNA-induced silencing complex (RISC), in which the argonaute (AGO) protein plays a pivotal role. The subsequent binding of the miRNA-RISC complex to its target mRNA instigates the degradation of the mRNA using the AGO, thereby repressing the associated gene expression (Li et al. 2018). Reinhart et al. (2002) detected the first-ever miRNA in plants. In addition to regulating numerous physiological processes, miRNAs also impact plants’ tolerance towards various abiotic stresses, including salinity stress. Several miRNAs are conserved for plant salt stress response (Islam et al. 2022). Studies show that miRNAs influence salt stress tolerance by either modulating hormonal pathways (Baek et al. 2016) or antioxidant systems of a plant (Cheng et al. 2021).

Flax (*Linum usitatissimum* L., family Linaceae), commonly known as flaxseed or linseed, is considered a multipurpose crop. It serves as a food, oilseed, and fibre plant. Flaxseed is one of the richest sources of lignans. The major lignans found in flax are secoisolariciresinol diglucoside (SDG; 294–2410 mg/100 g), secoisolariciresinol (SECO; 257.6 mg/100 g), lariciresinol (3.04–11.46 mg/100 g), matairesinol (0.55–6.68 mg/100 g) and pinoresinol (3.32 mg/100 g). However, considerable variation in lignan content has been observed among different flax genotypes (Goyal et al. 2014; Rodríguez-García et al. 2019). Flax is susceptible to various abiotic stress factors, including drought, salinity, and heat, which endanger the plant’s survival and reduce its overall productivity (Yadav et al. 2024). Since flax is both a great reservoir of lignans and susceptible to salinity stress, it was selected as the ideal study plant to correlate these two factors.

Lignans accumulate in plants and act primarily as defence substances against abiotic stresses. However, the lignan contents are dependent on the plant genotypes. Therefore, we searched for an answer to the question—“How much better can a flaxseed genotype with a high lignan content adapt to salinity stress than a flaxseed genotype with a low lignan content?”. Several morphological, biochemical, microscopic, and genomic expression assays were conducted to investigate the salinity stress responsiveness of two selected flaxseed genotypes, Flanders and Astella, which have contrasting lignan content. To the best of our knowledge, this is the first study on salt stress tolerance in a multi-utilitarian crop, such as flax, that focuses on lignan-related miRNAs.

## Materials and methods

### Plant materials and salt treatment

Seeds of flax genotypes Flanders and Astella were kindly provided by Agritec Plant Research Ltd. (Zemědělská, Šumperk, Czech Republic). We have shown that the main lignan (SDG) content is much higher in Astella (13.07 mg/g) than in Flanders (7.75 mg/g) (Harenčár 2023). Healthy and uniform seeds were placed on filter paper moistened with tap water and incubated for germination in a growth chamber (KK 400, POL-EKO, Wodzisław Śląski, Poland) at 23 ± 2 °C in the dark. One-week-old, healthy seedlings of uniform height were transferred to TS2 white peat potting substrate (Klasmann-Deilmann GmbH, Geeste, Germany) and kept in square pots measuring 8 cm × 8 cm × 9.5 cm (length × width × height). Each pot contained five seedlings. The pots were then incubated in the growth chamber at 23 ± 2 °C under a 16/8 h photoperiod, light intensity of 5000 lx provided by fluorescent tube lights, and a humidity level of 50%. The substrate was irrigated three times a week (on Mondays, Wednesdays, and Fridays) with a watering solution (Grandiol Universal Liquid Plant Food at a dilution of 1:20 with distilled water) up to a reference weight of 272 g (pot + substrate + watering solution), representing 80% of the maximum soil water-holding capacity (MSWC) of the substrate measured gravimetrically (Junker et al. 2015). The seedlings were allowed to grow for two more weeks.

Experimental treatments were arranged in a randomised complete block design. Three-week-old, randomly selected plants of both genotypes were subjected to treatment. The watering solution and an aqueous 100 mM NaCl (Sigma-Aldrich) solution were used for the control and salt stress treatments, respectively. The substrates were irrigated thrice a week by treatment solutions up to the MSWC of 80%. Plant samples were harvested after 1 week for the analyses.

### Morphological assays and growth parameter estimations

Various shoot, root, and leaf parameters were recorded. Due to the differential growth rates of Flanders and Astella, the data were processed in two steps to obtain unbiased results, as suggested by Julkowska and Testerink (2015). First, the relative changes were estimated by subtracting the values obtained before stress from those obtained after stress, until otherwise mentioned. Second, the data were normalised relative to the control treatments. The normalised relative data were plotted into graphs. The percentage (%) change of salt stress treatment data was calculated relative to the respective controls.

The shoot length (SL; cm) was measured using a ruler scale. The root traits, such as length (RL; cm), diameter (RD; cm), volume (RV; mm^3^), and the number of root tips (RT) and forks (RF), were measured using a scanner (Epson Expression 11000XL), calibrated and coupled with WinRHIZO Pro 2013e (Regent Instruments Inc.) software. The leaf number (LN) was counted.

The shoot and root FWs (SFW and RFW, respectively; mg) were estimated during harvest. The shoot and root DWs (SDW and RDW, respectively; mg) were measured after drying at 105 °C for 1 h and then at 80 °C for a further 4 h. Shoot and root relative water contents (SRWC and RRWC, respectively) were calculated according to Yan et al. (2023) using the following formulas:

$${\text{SRWC }}\left( \% \right) \, = \, \left[ {\left( {{\text{SFW }}{-}{\text{ SDW}}} \right) \, /{\text{ SFW}}} \right] \, \times { 1}00$$

$${\text{RRWC }}\left( \% \right) \, = \, \left[ {\left( {{\text{RFW }}{-}{\text{ RDW}}} \right) \, /{\text{ RFW}}} \right] \, \times { 1}00$$

The ratios between RL/SL, RFW/SFW, RDW/SDW, and RRWC/SRWC were calculated. Root mass density (RMD) was calculated using the formula:

$${\text{RMD }}\left( {{\text{mg}}/{\text{mm}}^{{3}} } \right) \, = {\text{ RFW }}/{\text{ RV}}$$

The leaf relative water content (LRWC) was measured according to Turner (1986). The first 15 leaves of a plant were removed using a fine-tipped scissor, and leaf fresh weight (LFW) was immediately recorded. The leaves were then floated on distilled water at 4 °C. After 24 h, leaf turgid weight (LTW) was determined. The leaves were then dried, and leaf dry weight (LDW) was recorded. LRWC was calculated using the following formula:

$${\text{LRWC }}\left( \% \right) \, = \, \left[ {\left( {{\text{LFW }}{-}{\text{ LDW}}} \right)/\left( {{\text{LTW }}{-}{\text{ LDW}}} \right)} \right] \, \times { 1}00$$

### Reactive oxygen species (ROS) generation assay

The hydrogen peroxide (H_2_O_2_) concentration was determined according to Sinha (1972) to assess the extent of ROS generation. A mixture containing 1 g of leaf sample, 100 mg of activated charcoal, and 10 ml of 5% trichloroacetic acid (TCA) was centrifuged (ROTINA 420 R; Hettich, Tuttlingen, Germany) at 15,617 *g* for 10 min. The resulting supernatant (0.5 ml) was mixed with 0.01 M phosphate buffer solution (pH 7.0) to make a final volume of 1 ml, to which a 3 ml mixture of potassium dichromate and glacial acetic acid (1:3, w/v) was added. The H_2_O_2_ concentration (μmol/g FW) was estimated by spectrophotometric absorption (Jenway 6405 UV/Vis spectrophotometer; Stone, Staffordshire, UK) of the final mixture at *A*_*570nm*_ against 10–160 μmol standards.

### Lipid peroxidation assays

The malondialdehyde (MDA) assay was used to estimate membrane lipid peroxidation under salt stress-derived oxidative damage. The MDA content was measured using the thiobarbituric acid (TBA) method according to Dhindsa et al. (1981). In brief, a mixture containing 0.1 g of leaf sample, 0.5% TBA, and 20% TCA was heated in a hot water bath at 90 °C for 30 min, and then cooled in an ice bath. The supernatant was then extracted by centrifugation at 15,617 *g* for 20 min. The MDA content (nmol/g FW) was estimated by spectrophotometric absorption of the supernatant at *A*_*532nm*_ using an extinction coefficient of 155^–1^ mM^−1^ cm^−1^.

### Enzymatic antioxidants analyses

#### Sample preparation

The freshly harvested leaves were homogenised in a chilled 0.1 M potassium phosphate buffer solution (pH 7.8). The homogenate was centrifuged at 4 °C, 15,617 *g* for 20 min. The resulting supernatant was stored at − 80 °C to analyse the enzymatic activities.

#### Superoxide dismutase (SOD)

The SOD activity was measured according to Giannopolitis and Ries (1977). The 3.7 ml reaction mixture contained 100 ml of the plant supernatant, 0.5 mM nitroblue tetrazolium (NBT), 17 mM methionine, 0.1 mM EDTA, 60 μM riboflavin, and 0.1 M potassium phosphate buffer (pH 7.8). The one unit of SOD activity (U/mg protein) was defined as the quantity of enzyme required to inhibit the photolytic reduction of NBT by 50% and was estimated by spectrophotometric absorption at *A*_*560nm*_.

#### Catalase (CAT)

The CAT activity was assayed according to Aebi (1984). The 1 ml reaction mixture contained 20 μl of the plant supernatant, 10 mM H_2_O_2_, and 0.05 M potassium phosphate buffer (pH 7.0). To express the CAT activity (U/g protein), the decomposition of H_2_O_2_ was recorded by the decrease in spectrophotometric absorbance at *A*_*240nm*_ per unit time.

#### Ascorbate peroxidase (APX)

The Nakano and Asada (1987) method was used to estimate APX activity. The 1 ml reaction mixture contained 100 ml of the plant supernatant, 1.5 mM H_2_O_2_, 0.5 mM ascorbic acid, 0.1 mM EDTA, and 0.05 M potassium phosphate buffer (pH 7.0). The enzyme activity (U/g protein) was determined by measuring the oxidation rate of ascorbic acid, which was monitored by the rate of decrease in spectrophotometric absorbance at* A*_*290nm*_ per min.

#### Guaiacol peroxidase (GPOX)

The GPOX activity was measured according to Pütter (1974). The reaction mixture contained 100 μl of the plant supernatant, 50 μl of guaiacol solution, 30 μl of H_2_O_2_, and 3 ml of 3 M phosphate buffer (pH 7.0). The increase in absorbance of the mixture at* A*_*436nm*_ was recorded, and GPOX activity (U/g protein) was calculated using an extinction coefficient of 6.39 cm^2^/μmol.

### Non-enzymatic antioxidant analyses

#### Sample preparation

The freshly harvested 0.10 g of leaves was extracted with 10 ml of 80% ethanol for 2 h. After centrifugation at 1789 g (Rotofix 32 A, Hettich) for 10 min, the supernatant was used for the following experiments.

#### Total polyphenol (PP) content

The total PP content was measured according to Singleton and Rossi (1965). The reaction mixture containing 0.1 ml of the plant supernatant, 0.1 ml of the Folin–Ciocalteu reagent, 1 ml of 20% (w/v) sodium carbonate solution, and 8.8 ml of distilled water was left in the dark for 30 min. Following incubation, the absorbance of the mixture at* A*_*700nm*_ was measured spectrophotometrically. Gallic acid (GA; 25–300 mg/l, coefficient of determination *R*^*2*^ = 0.998) was used as a standard, and the results were expressed in mg/l of GA equivalents (GAE).

#### Total phenolic acid (PA) content

The total PA content was determined according to Jain et al. (2017). The reaction mixture contained 0.5 ml of the plant supernatant, 0.5 ml of 0.5 M hydrochloric acid, 0.5 ml of the Arnova reagent (10% NaNO_2_ + 10% Na_2_MoO_4_), 0.5 ml of 1 M sodium hydroxide (w/v), and 0.5 ml of water. The absorbance of the mixture at* A*_*490nm*_ was measured spectrophotometrically. Caffeic acid (CA; 1–200 mg/l, coefficient of determination *R*^*2*^ = 0.999) was used as a standard, and the results were expressed in mg/g of CA equivalents (CAE).

#### Radical scavenging activity

The radical-scavenging activity of samples was measured using 2,2-diphenyl-1-picrylhydrazyl (DPPH) (Sanchéz-Moreno et al. 1998). The reaction mixture contained 0.4 ml of the plant supernatant and 3.6 ml of DPPH solution (0.025 g of DPPH in 100 ml of ethanol). The absorbance of the mixture was determined spectrophotometrically at* A*_*515nm*_. Trolox (6-hydroxy-2,5,7,8-tetramethylchroman-2-carboxylic acid) (10–100 mg/l, coefficient of determination *R*^*2*^ = 0.989) was used as a standard, and the results were expressed in mg/g Trolox equivalents (TEAC).

### Proline content analysis

The proline content was determined according to Bates et al. (1973). The leaf tissues were homogenised in 3% (w/v) sulfosalicylic acid and reacted with an equal volume of acid ninhydrin and glacial acetic acid for 1 h at 95 °C in a water bath. The reaction mixture was then transferred to an ice bath to terminate the reaction. After 10 min, toluene was added to each tube and shaken. The proline content (μmol/g FW) was estimated by spectrophotometric absorbance of the mixture at *A*_*520nm*_.

### RNA isolation

Total RNA was isolated from 100 mg of shoot samples using the NucleoSpin RNA Plant mini kit (Macherey–Nagel) according to the kit-specified protocol. The isolated RNA was eluted in 50 μl of kit-supplied RNase-free water. The quantity and purity of the total RNA were estimated using a spectrophotometer (NanoPhotometer P 360, Implen).

### Two-tailed qPCR (TT-qPCR) assay

Expression analysis of miRNA genes was performed using the TT-qPCR Assay kit (BioVendor – Laboratorni medicina a.s., Karasek, Brno, Czech Republic), adopting the protocol of Androvic et al. (2017). The protocol of the kit is biphasic. BioVendor designed the primer sets for three miRNA genes (miR168a, miR399g and miR828a) for both phases based on flax miRNA sequences provided by the miRBase database (https://www.mirbase.org/), and these were supplied with the kit. In the first phase, cDNA was synthesised from total RNA using the C1000 thermal cycler with a dual 48/48 fast reaction module (Bio-Rad). An equal volume of total RNA (326 ng) was used for all reactions. In the second phase, qPCR was performed using the C1000 thermal cycler with CFX96 Real-Time System optical reaction module (Bio-Rad). The expressions of cyclophilin and the eukaryotic transcription initiation factor 5A (EIF5A) (primers, Table S1) were used to normalise the tested miRNA expression levels. The specificity of the qPCR products was determined by the melting curve (Cq) analysis using CFX Maestro 2.0 version 5.0.021.0616 (Bio-Rad). Data were processed according to Livak and Schmittgen (2001) and expressed as the fold change.

### Viability assay of flax roots

The viability of flax root cells was determined by co-staining with fluorescein diacetate (FDA; Thermo Fisher Scientific) and propidium iodide (PI; Sigma-Aldrich) according to Jones et al. (2016) with a few modifications. The FDA and PI were used to stain live (green fluorescence) and dead (red fluorescence) cells, respectively. Stock solutions were prepared by dissolving 5 mg of FDA and 1 mg of PI in 1 ml of acetone and 1 ml of water, respectively, and stored at  – 20 °C. The working FDA-PI co-staining solution was prepared freshly by mixing 4 μl of FDA stock solution and 10 μl of PI stock solution to a final volume of 1 ml in water. Root samples were dipped in the staining solution and incubated for 5 min in the dark. Afterwards, the samples were observed under the Leica DM5500B fluorescence microscope equipped with a Leica DFC450 C digital camera (Leica Microsystems). The filter sets L5 (BP 480/40, DM 505, BP 527/30) and N2.1 (BP 515–560, DM 580, LP 590) were used to detect FDA and PI staining, respectively. The images were processed with ImageJ version 1.54d (Schneider et al. 2012).

### Statistical analysis

All experiments were performed in three biological and three technical replicates. Data were analysed by one-way analysis of variance (ANOVA) to calculate statistical significance. Significant differences in treatment data (mean ± standard error) were estimated using Duncan’s multiple range test (DMRT) at *P* < 0.05 significance level (Duncan 1955). Both analyses were performed in SPSS version 19 (IBM Corporation). Graphs were prepared in OriginPro 2021 version 9.8.0.200 (OriginLab Corporation). The independent variable data were logarithmically transformed, standardised, and normalised for multivariate analysis using Pearson’s correlation coefficient analysis (PCC), principal component analysis (PCA), and heatmap generation. The multivariate analysis was executed in R version 4.4.2 (R Core Team 2024) coupled with RStudio version 2024.09.1 Build 394 (Posit Team 2024).

## Results

### Effect of salinity stress on plant morphology

**Fig. 1:**
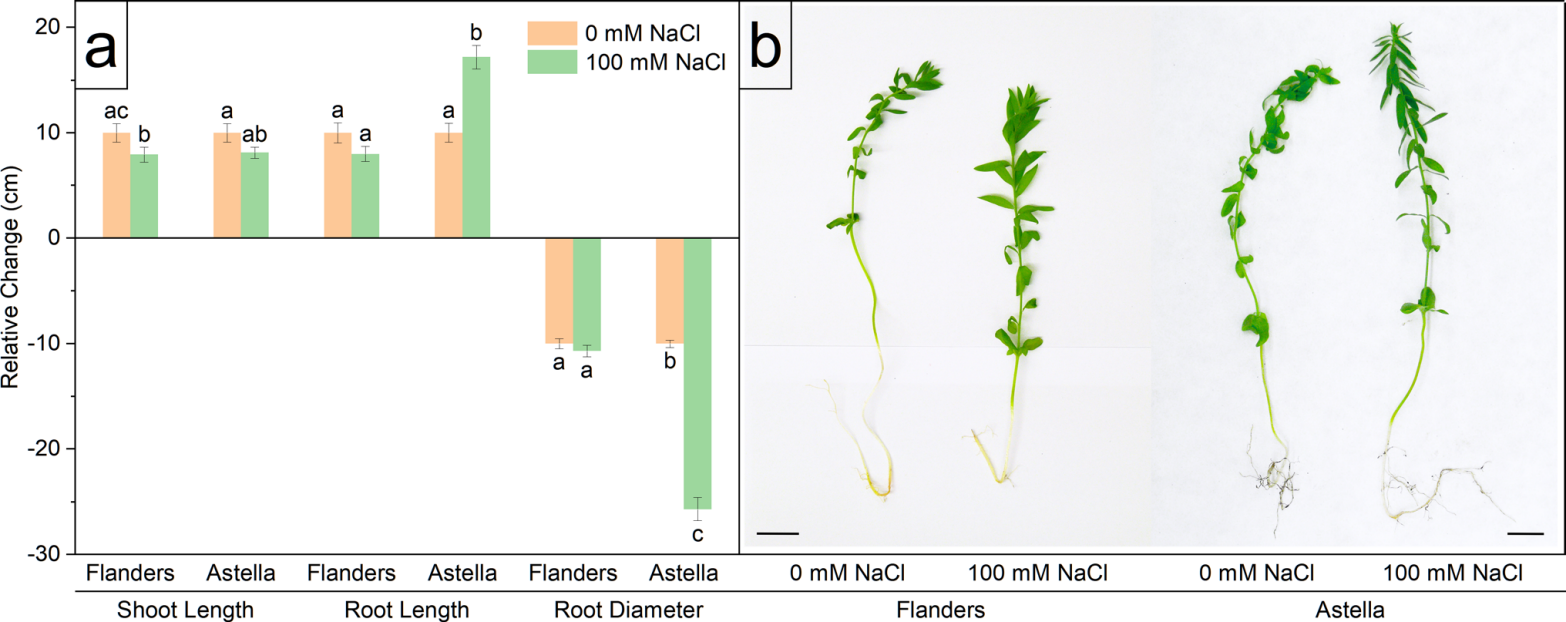
Changes in morphological parameters of two flax genotypes, Flanders and Astella, under 1 week of 100 mM NaCl stress. The data represent relative changes during the stress period. Data were normalised to the control treatment. **a** Shoot length, root length, and diameter. **b** A photograph depicting comparative morphology between Flanders and Astella in control and stressed conditions. Scale bar = 10 mm. Data represent mean ± standard error (*n* = 3). Different letters followed by the means represent statistical differences at *P* < 0.05 (one-way ANOVA with DMRT post hoc test)

**Fig. 2:**
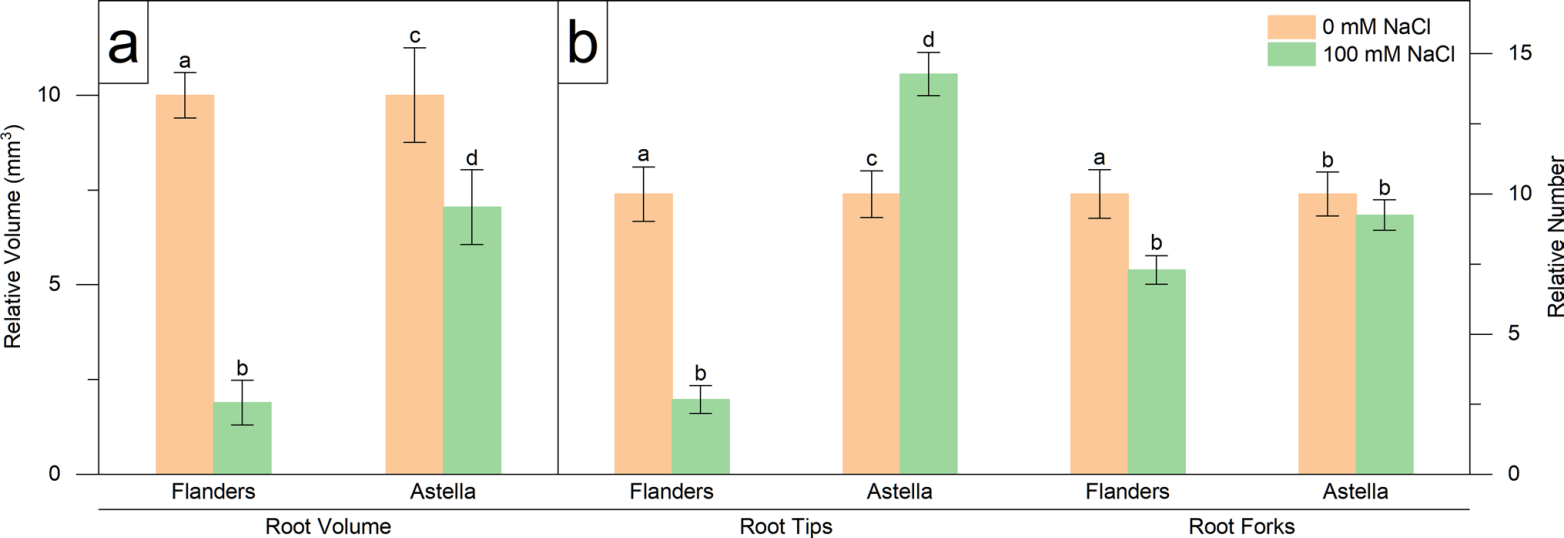
Changes in morphological parameters of two flax genotypes, Flanders and Astella, under 1 week of 100 mM NaCl stress. The data represent relative changes during the stress period. Data were normalised to the control treatment. **a** Root volume. **b** Root tips and forks. Data represent mean ± standard error (*n* = 3). Different letters followed by the means represent statistical differences at *P* < 0.05 (one-way ANOVA with DMRT post hoc test)

**Fig. 3:**
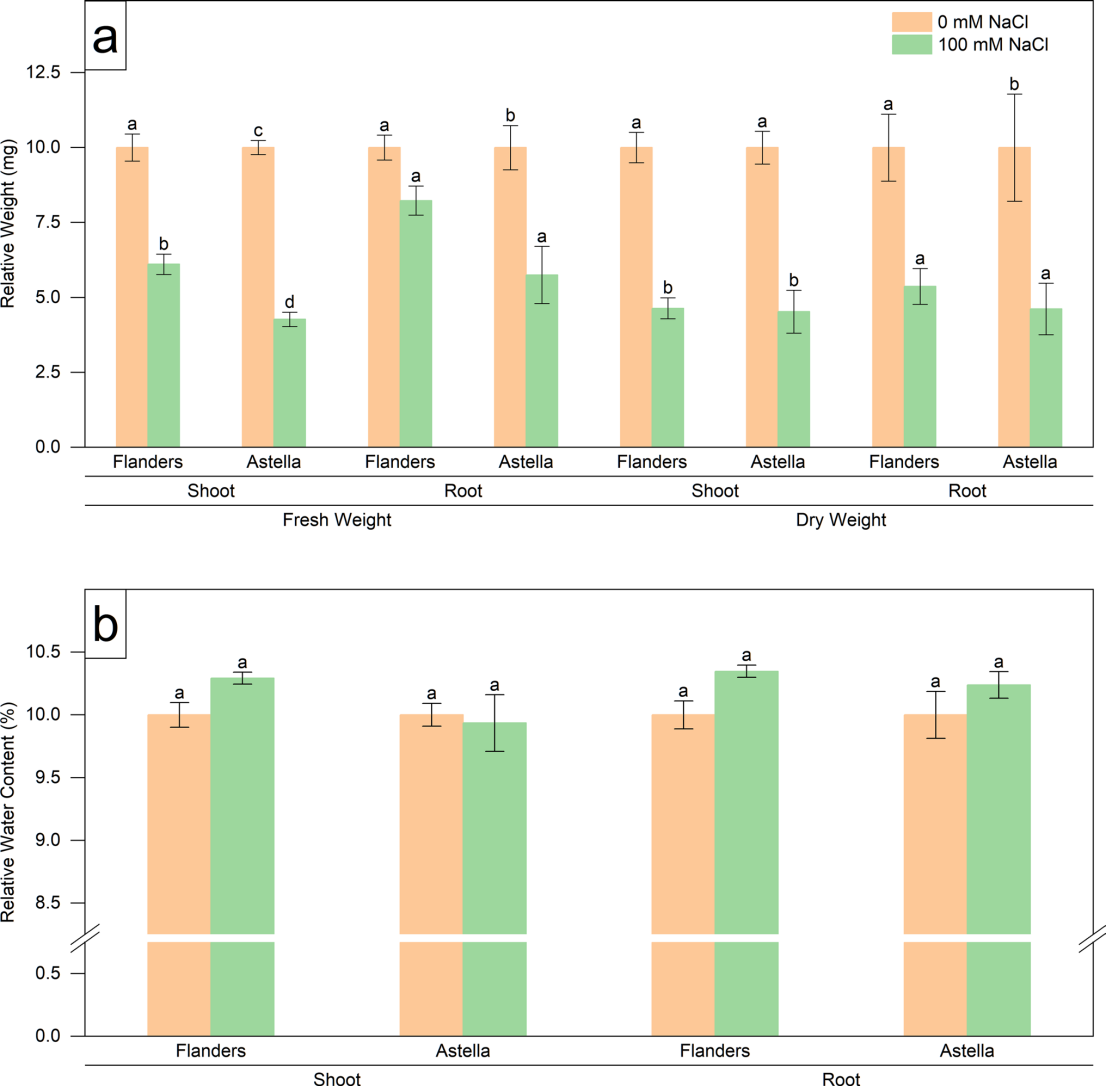
Changes in morphological parameters of two flax genotypes, Flanders and Astella, under 1 week of 100 mM NaCl stress. The data represent relative changes during the stress period. Data were normalised to the control treatment. **a** Fresh and dry weights of shoots and roots. **b** Relative water contents of shoot and root. Data represent mean ± standard error (*n* = 3). Different letters followed by the means represent statistical differences at *P* < 0.05 (one-way ANOVA with DMRT post hoc test)

**Fig. 4:**
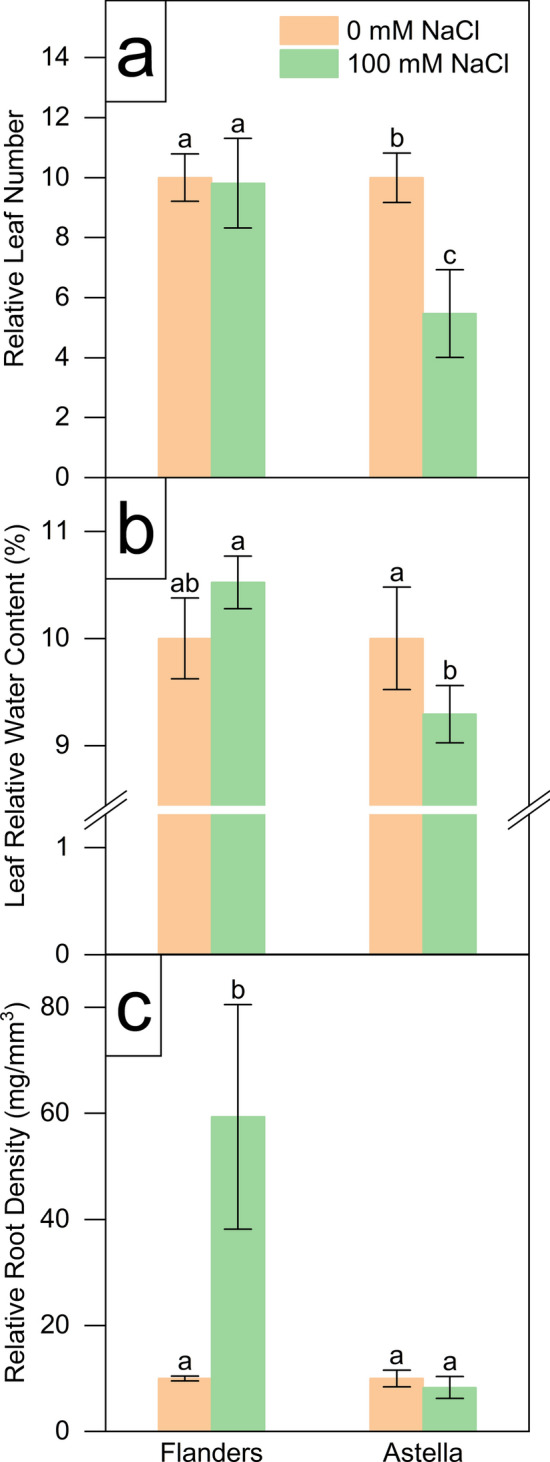
Changes in morphological parameters of two flax genotypes, Flanders and Astella, under 1 week of 100 mM NaCl stress. The data represent relative changes during the stress period, except for leaf relative water content at the end of NaCl stress. Data were normalised to the control treatment. **a** Leaf number. **b** Leaf relative water content. **c** Root density. Data represent mean ± standard error (*n* = 3). Different letters followed by the means represent statistical differences at *P* < 0.05 (one-way ANOVA with DMRT post hoc test)

The 7-day exposure to 100 mM NaCl salt stress did not result in lethality for either Flanders or Astella flax genotypes. However, their differential responses to salt stress were evident from several morphological assessments (Figs. 1a, 2, 3, 4; Table 1; Table S2) and visual perspectives (Fig. 1b).

**Table 1:** Ratio of different shoot and root parameters of two flax genotypes, Flanders and Astella, under 1 week of 100 mM NaCl stress

Genotype	RL/SL	RFW/SFW	RDW/SDW	RRWC/SRWC
Flanders	1.01	1.35	1.16	1.01
Astella	2.12	1.35	1.02	1.03

The data were estimated as relative changes under the stress period, and normalised against the control treatment. The ratios of normalised relative values are represented in this table. The ratios of control treatment values are omitted. *R* root, *S* shoot, *L* length, *DW* dry weight, *FW* fresh weight, *RWC* relative water content

The impact of salt stress on the visual growth parameters of both genotypes was observed. In comparison with the control, shoot length (SL), root volume (RV), and the number of root tips (RT) and root forks (RF) were reduced in salinity-treated Flanders. Conversely, salinity treatment in Astella increased root length (RL) and RT, and decreased root diameter (RD) and RV compared to the control. Salinity treatment reduced RL, RD, RV, and RT in Flanders over Astella (Figs. 1a, 2a, b; Table S2). The comparatively higher RL/SL ratio of Astella also demonstrated that its roots grew much faster than the shoots under NaCl stress than those of Flanders (Table 1). Consequently, the visual appearance of salt-stressed Flanders plants was stunted among the other treatment plants (Fig. 1b).

The shoot fresh weight (SFW), shoot dry weight (SDW), root fresh weight (RFW), and root dry weight (RDW) were analysed in Flanders and Astella to investigate the changes in plant biomass in response to salt stress. The NaCl stress significantly decreased biomass, apart from RFW and RDW of Flanders. This finding indicated that salinity affected Flanders roots less (Fig. 2a; Table S2). The equal RFW/SFW ratio between the two genotypes indicated that their stress-mediated FW change was similar. Conversely, a higher RDW/SDW ratio in Flanders exhibited a more pronounced RDW increase than in Astella under salt stress conditions (Table 1). The findings suggested that the Flanders RDW exhibited less salinity sensitivity than Astella.

The leaf number (LN) of NaCl-treated Astella was significantly reduced compared to its control and NaCl-treated Flanders (Fig. 4a; Table S2). There was no significant change in salt stress-mediated shoot relative water content (SRWC) or root relative water content (RRWC) (Fig. 3b; Table S2), and the ratio of root relative water content (RRWC) to shoot relative water content (SRWC) was almost equal (Table 1). It indicated a similar water content change in the shoots and roots of the salinity-treated plants of both genotypes. Notably, leaf relative water content (LRWC), a vital marker of salt stress, exhibited a significant decrease in the NaCl-treated Astella compared to its control and NaCl-treated Flanders (Fig. 4b; Table S2). A significant increase in root mass density (RMD) in Flanders compared to Astella under stress was also observed (Fig. 4c; Table S2).

### Effect of salinity stress on ROS generation and cell damage

**Fig. 5:**
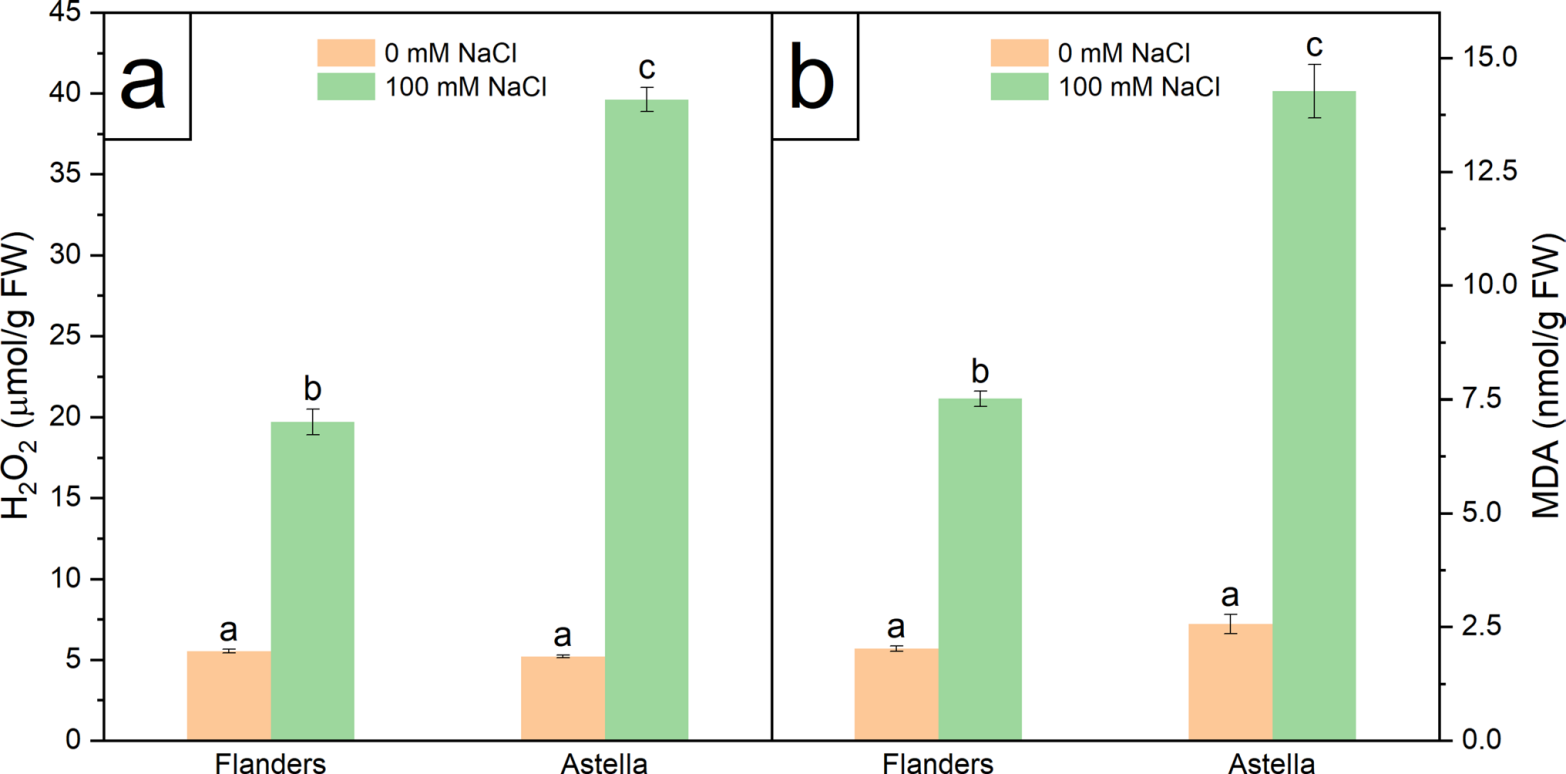
Biochemical estimation of ROS and associated cell damage in two flax genotypes, Flanders and Astella, after cultivation for 1 week under 100 mM NaCl stress. **a** H_2_O_2_, a ROS marker. **b** MDA content, a cell damage marker. The ROS is a regular byproduct of cellular metabolism. Antioxidants scavenge ROS molecules to create a neutral redox potential inside the cell. Overproduction of ROS during salt stress disturbs the redox state of the affected cells, generating the oxidative burst situation. Excessive ROS molecules damage lipid bilayer membranes by lipid oxidation, generating MDA as the end-product. Data represent mean ± standard error (*n* = 3). Different letters followed by the means represent statistical differences at *P* < 0.05 (one-way ANOVA with DMRT post hoc test). *MDA* malondialdehyde

The cellular extent of salt stress-derived ROS generation and subsequent damage was estimated by H_2_O_2_ and lipid peroxidation assays, respectively. MDA is the final product of lipid peroxidation and is generally considered an indicator of oxidative stress-mediated cell membrane damage. Together, H_2_O_2_ and MDA are salt stress markers (Soltabayeva et al. 2021). In the present study, NaCl treatment significantly increased the amount of salt stress markers in the studied plants. The induction was higher in Astella than in Flanders (Fig. 5a, b; Table S3). These results suggested that Astella experienced more salt toxicity than Flanders.

### Effect of salinity stress on antioxidant system

Cellular stress activates antioxidant enzymes in plants. In this study, genotype-dependent significant changes in the levels of several enzymatic and non-enzymatic antioxidant markers were identified in Flanders and Astella upon NaCl stress treatment.

**Fig. 6:**
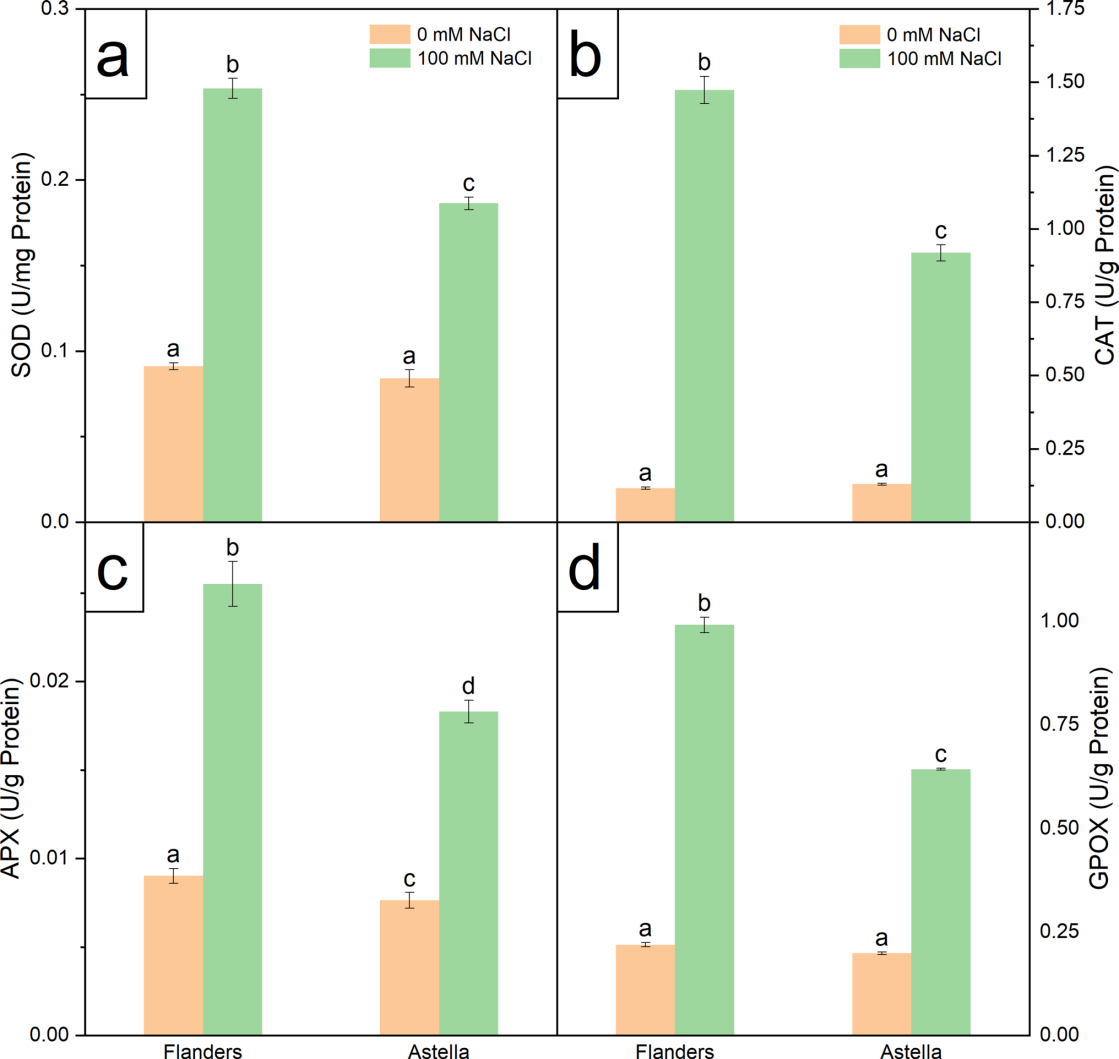
Biochemical estimation of enzymatic antioxidants in two flax genotypes, Flanders and Astella, after cultivation for 1 week under 100 mM NaCl stress. In response to the salt stress-mediated oxidative burst, plants activate a well-synchronised antioxidant system for ROS scavenging. **a** The frontline defence is provided by SOD through the conversion of superoxide (O_2_^•–^) to H_2_O_2_. The **b** CAT, **c** APX, and **d** GPOX convert H_2_O_2_ to H_2_O and O_2_. Data represent mean ± standard error (*n* = 3). Different letters followed by the means represent statistical differences at *P* < 0.05 (one-way ANOVA with DMRT post hoc test). *APX* ascorbate peroxidase, *CAT* catalase, *GPOX* guaiacol peroxidase, *SOD* superoxide dismutase

**Fig. 7:**
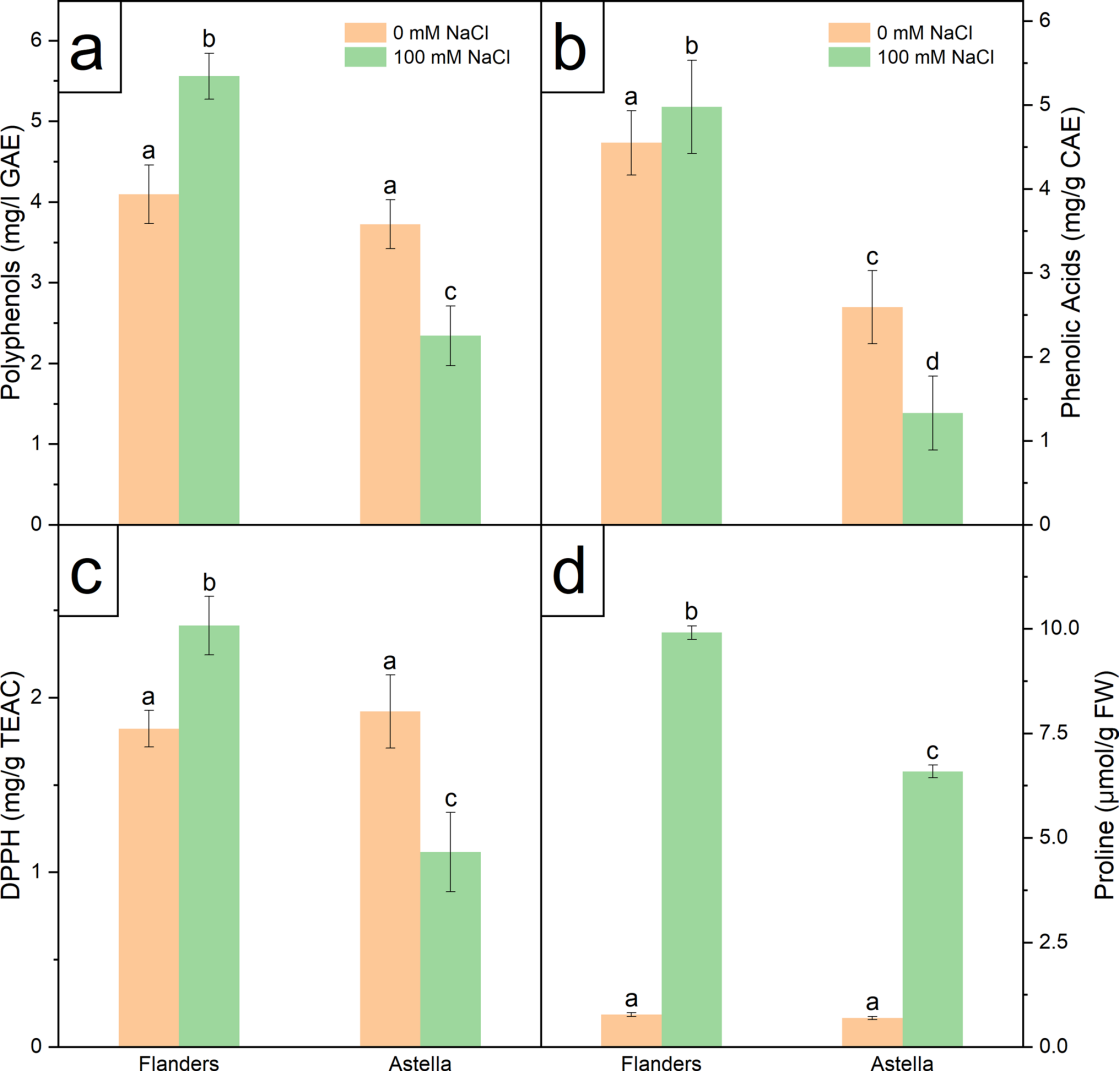
Biochemical estimation of non-enzymatic antioxidants, total radical-scavenging activity, and proline content in two flax genotypes, Flanders and Astella, after 1 week of 100 mM NaCl stress. The non-enzymatic antioxidants, polyphenols (**a**) and phenolic acids (**b**), are excellent antioxidants due to the presence of multiple hydroxyl groups. **c** The total radical-scavenging activity was measured using the DPPH assay. **d** Proline provides cellular protection against salt stress in numerous ways. Data represent mean ± standard error (*n* = 3). Different letters followed by the means represent statistical differences at *P* < 0.05 (one-way ANOVA with DMRT post hoc test). *CAE* caffeic acid equivalent, *DPPH* 2,2-diphenyl-1-picrylhydrazyl, *GAE* gallic acid equivalent, *TEAC* 6-hydroxy-2,5,7,8-tetramethylchroman-2-carboxylic acid

Salt stress elevated the levels of critical enzymatic antioxidant markers (SOD, CAT, APX, and GPOX) and two crucial non-enzymatic antioxidant markers (PP and PA) in Flanders over Astella (Figs. 6, 7a, b; Table S3). The total radical-scavenging activity of antioxidants estimated by DPPH assay was higher in Flanders than in Astella under NaCl stress (Fig. 7c; Table S3).

Proline is a vital component of the cellular stress mitigation system, contributing to osmoregulation and antioxidant activity. The NaCl-treated plants of both genotypes had higher proline accumulation than the respective controls. Furthermore, salinity stress increased proline content more in Flanders than in Astella (Fig. 7d; Table S3). The results demonstrated genotype-dependent antioxidant activity under NaCl stress, with higher activity observed in Flanders than in Astella.

### Effect of salinity stress on lignan-related miRNAs

**Fig. 8:**
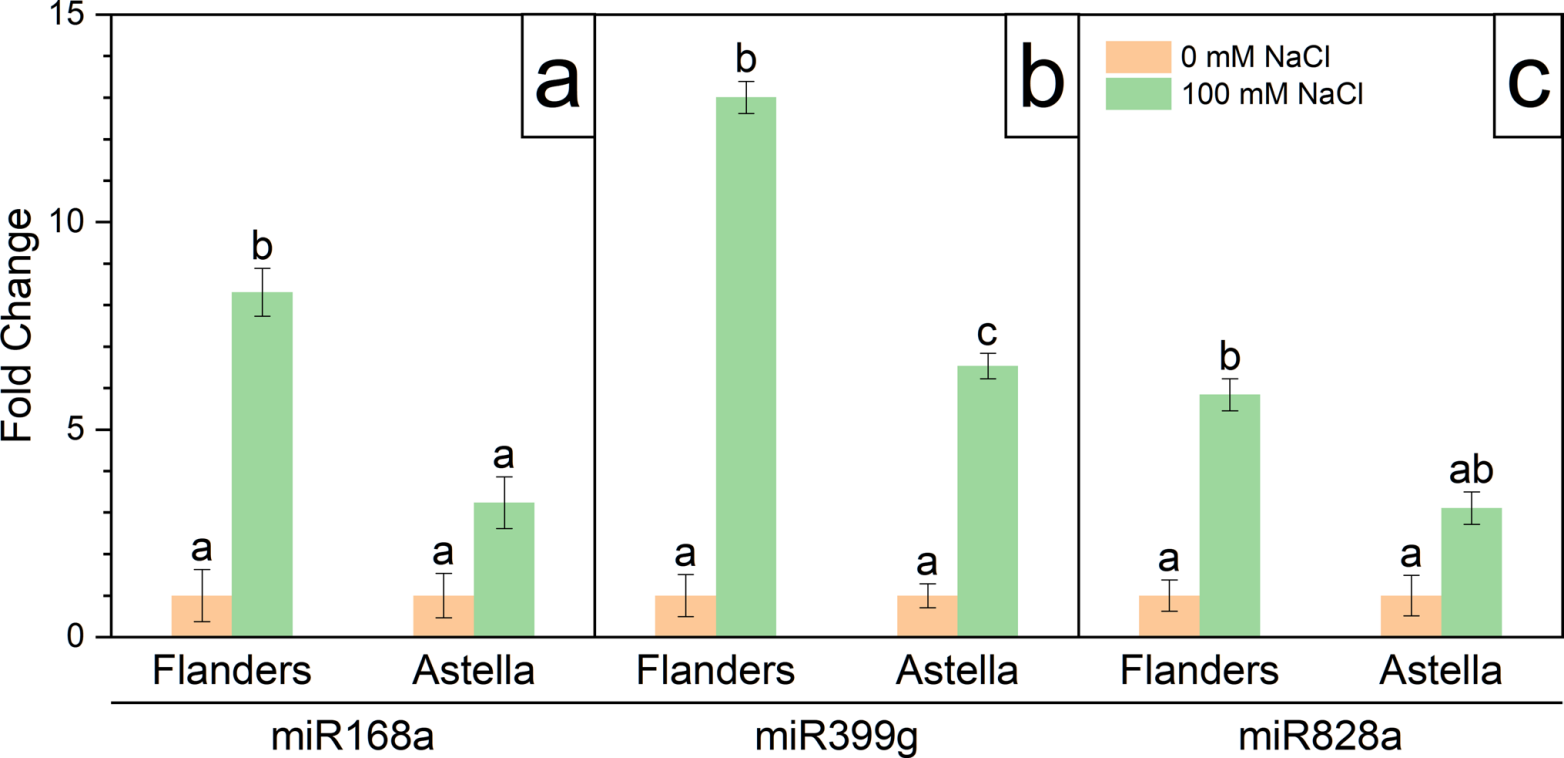
Genomic expression analysis of lignan-biosynthetic miRNAs in two flax genotypes, Flanders and Astella, after 1 week of 100 mM NaCl stress. The miRNAs were isolated from treatment plants, and the relative expressions of miR168a (**a**), miR399g (**b**), and miR828a (**c**) were measured using the two-tailed qPCR assay kit using cyclophilin and EIF5A as internal controls for normalisation. Data represent means ± standard errors (*n* = 3). Different letters followed by the means represent statistical differences at *P* < 0.05 (one-way ANOVA with DMRT post hoc test). *EIF5A* eukaryotic transcription initiation factor 5A

In this experiment, the two-tailed qPCR assay was performed to monitor the expression of three miRNA molecules, miR168a, miR399g, and miR828a. The study revealed that all the tested miRNA molecules were upregulated in response to NaCl stress in both genotypes. The expression levels of these miRNAs were higher in salt-stressed Flanders than in Astella (Fig. 8a–c).

### Effect of salt stress on root cell viability

**Fig. 9:**
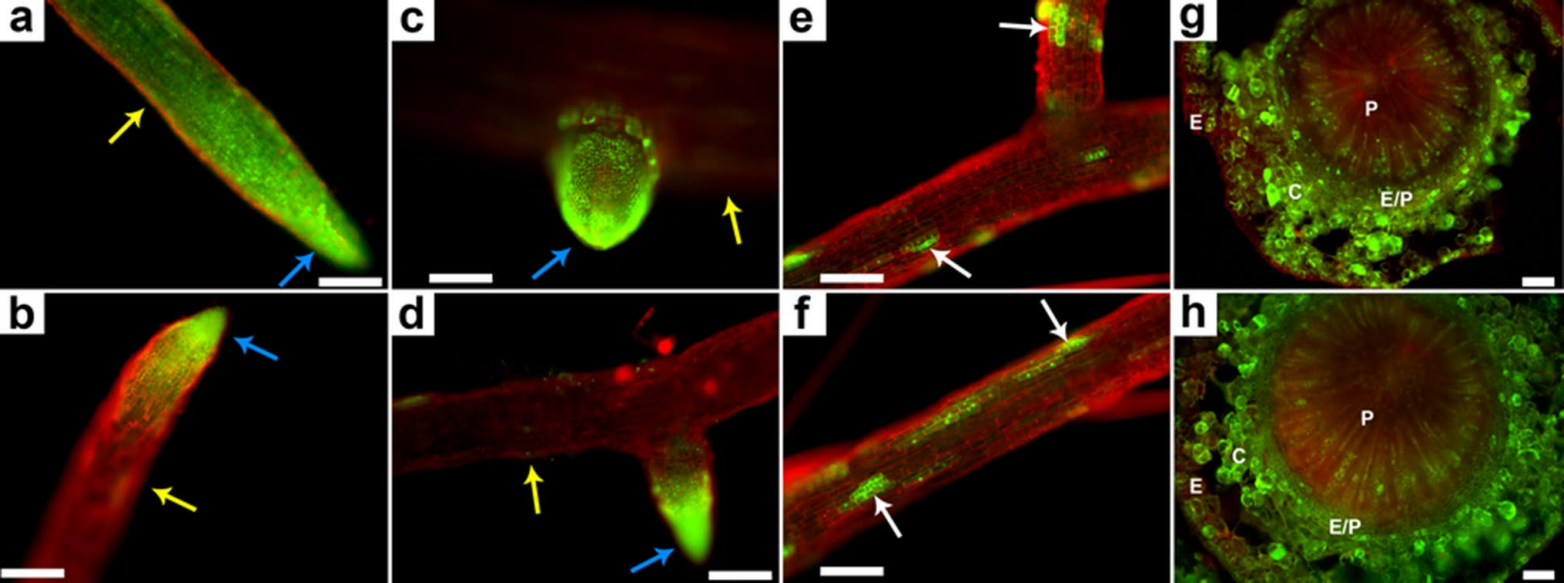
Fluorescence microscopic study of root cell viability of flax genotypes, Flanders and Astella, using FDA-PI differential staining after 1 week of 100 mM NaCl stress. Live cells were green and dead cells were red. **a**, **c**, **e**, and **g** Flanders roots. **b**, **d**, **f**, and **h** Astella roots. Root tips (blue arrows in **a** and **b**) and lateral root buds (blue arrows in **c** and **d**) were live in both genotypes. The root epidermal layer was mostly dead (yellow arrows in **a**, **b**, **c**, and **d**). A sporadic pattern of live cells was found in the root maturation zone (white arrows in **e** and **f**). **g**, **h** Cross-sectional views also depicted the dead epidermal layer (E). Scale bar = 200 µm. *C *cortex, *E* epidermis, *E/P* endodermis/pericycle, *P* pith

The viability assay of NaCl-treated flax roots was conducted using fluorescence microscopy. Through differential FDA-PI co-staining of root tissues, live and dead cells of root tissues appeared green and red, respectively. In both genotypes, root tips (Fig. 9a, b) and developing lateral root buds (Fig. 9c, d) appeared viable; however, the epidermal cells and the higher regions of the roots were non-viable. A sporadic pattern of viable cells was observed in the root maturation zone (Fig. 9e, f). Root cross-sectional views showed thriving root tissues despite the damaged epidermis layers (Fig. 9g, h).

### Multivariate analysis

**Fig. 10:**
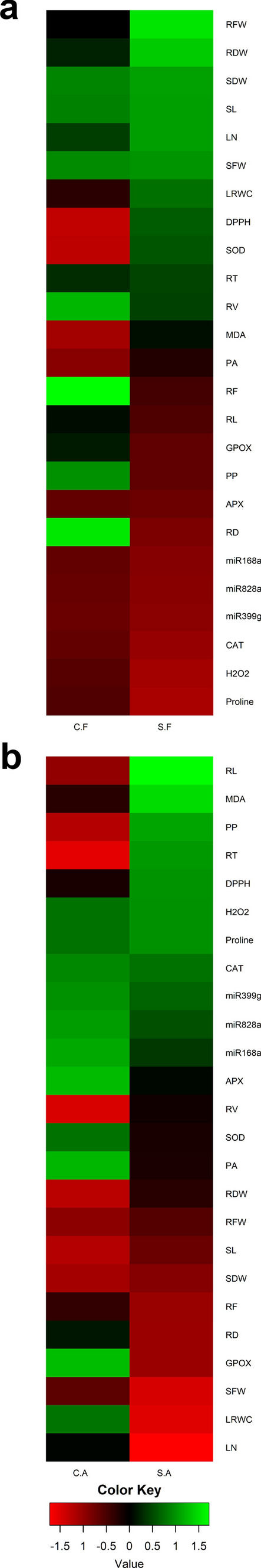
Heatmap analysis using the tested independent variables. Data were obtained after stressing two flax genotypes, Flanders and Astella, with 100 mM NaCl for 1 week. Data of 26 independent variables were logarithmically transformed, standardised, and normalised for this analysis. This analysis represents the abundance of the tested variables in the treated individuals (control treatments, C.F and C.A; salinity treatments, S.F and S.A). *APX* ascorbate peroxidase, *CAT* catalase, *D* diameter, *DPPH* 2,2-diphenyl-1-picrylhydrazyl, *DW* dry weight, *F* fork, *FW* fresh weight, *GPOX* guaiacol peroxidase, *L* length, *LN* leaf number, *LRWC* leaf relative water content, *MDA* malondialdehyde, *PA* phenolic acids, *PP* polyphenols, *R* root, *S* shoot, *SOD* superoxide dismutase, *T* tip, *V* volume

The data of 26 independent variables were linearised by logarithmic transformation, standardised to ensure the mean of each treatment was set to 0 with a standard deviation of 1, and normalised to create a data matrix with uniform distribution. The heatmap demonstrated the abundance of the tested variables in the control (C.F and C.A) and treated (S.F and S.A) samples (Fig. 10). The salt stress markers H_2_O_2_ and MDA were more abundant in salt-treated Astella (S.A) than in Flanders (S.F). In S.F, all tested variables, except proline, were more abundant than H_2_O_2_. Furthermore, several morphological markers (RFW, RDW, SDW, SL, LN, SFW, LRWC, RT, and RV) and antioxidant markers (DPPH and SOD) exhibited higher levels of abundance than MDA in S.F. In S.A, RL, PP, RT, and DPPH were more abundant than H_2_O_2_; RL was more abundant than MDA. The heatmap analysis revealed a differential stress regulation between Flanders and Astella.

**Fig. 11:**
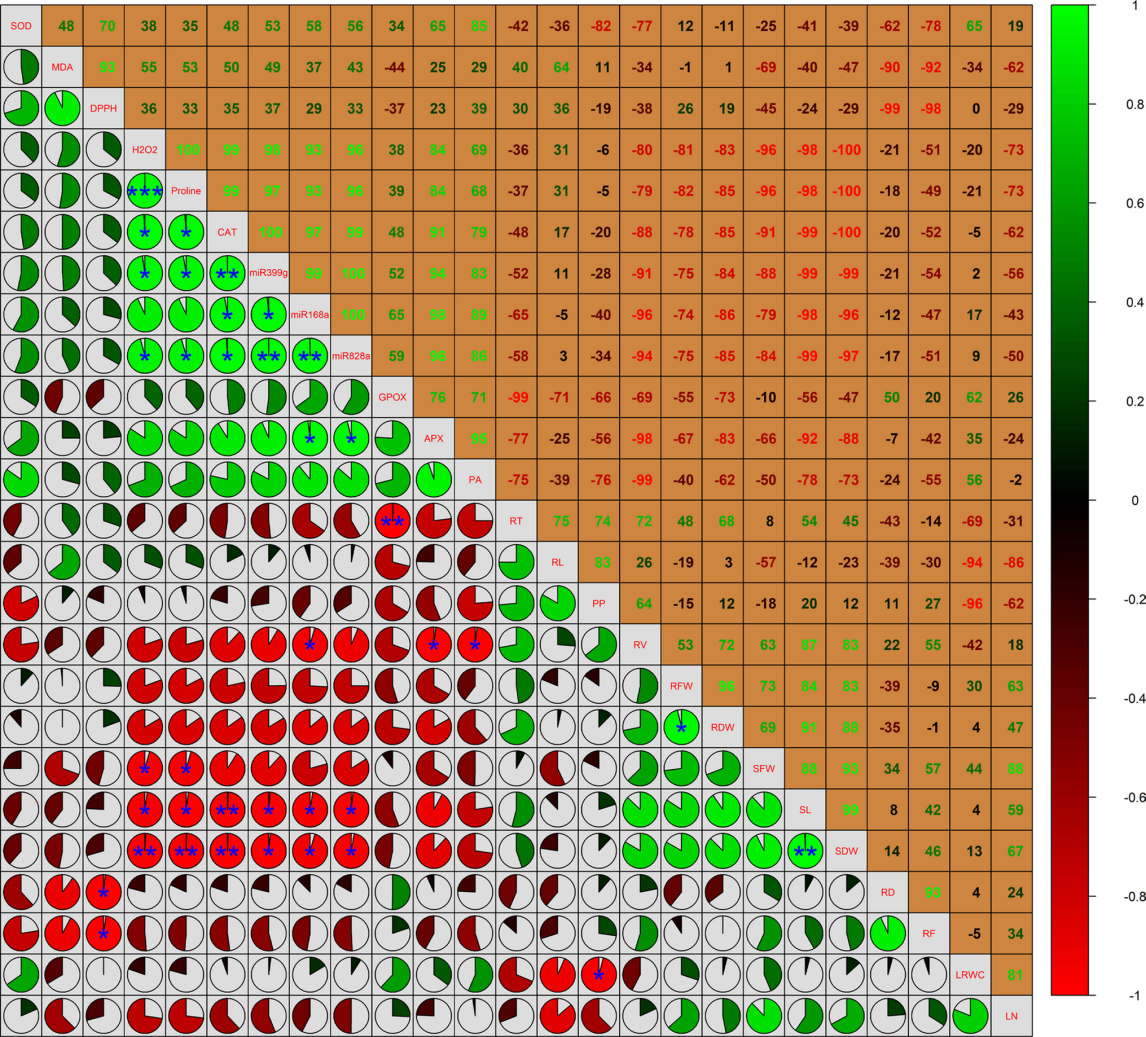
Pearson’s correlation coefficient analysis using the tested independent variables. Data were obtained after stressing two flax genotypes, Flanders and Astella, with 100 mM NaCl for 1 week. Data of 26 independent variables were logarithmically transformed, standardised, and normalised for this analysis. This analysis represents the correlation constants as pie figures and the percentage of the correlation values as numbers in the plot. *APX* ascorbate peroxidase, *CAT* catalase, *D* diameter, *DPPH* 2,2-diphenyl-1-picrylhydrazyl, *DW* dry weight, *F* fork, *FW* fresh weight, *GPOX* guaiacol peroxidase, *L* length, *LN* leaf number, *LRWC* leaf relative water content, *MDA* malondialdehyde, *PA* phenolic acids, *PP* polyphenols, *R* root, *S* shoot, *SOD* superoxide dismutase, *T* tip, *V* volume

The PCC demonstrated the overall correlation of the variables used in this study. Different correlation coefficient (*r*) thresholds were annotated according to Ratner (2009). It was observed that H_2_O_2_ had a high positive correlation (*r* ≥ 0.7) with proline, CAT, APX, and miRNAs and a high negative correlation (*r* ≤ -0.7) with morphological markers such as LN, RV, RFW, RDW, SFW, SL, and SDW. The MDA exhibited a high positive correlation (*r* ≥ 0.7) with DPPH and a high negative correlation (*r* ≤ -0.7) with RD and RF (Fig. 11; Table 2, S4). Lignans are a type of PP. Interestingly, H_2_O_2_ and MDA exhibited a weak correlation with PP in the present study (|*r*|≤ 0.3).

**Table 2:** Pearson’s correlation of different independent variables with H_2_O_2_ and MDA

Variables	H_2_O_2_		Variables	MDA	
	Correlation	Annotation		Correlation	Annotation
H_2_O_2_	+ + + +	Perfect	MDA	+ + + +	Perfect
Proline	+ + +	High	DPPH	+ + +	High
CAT	+ + +	High	RL	+ +	Moderate
miR399g	+ + +	High	H_2_O_2_	+ +	Moderate
miR828a	+ + +	High	Proline	+ +	Moderate
miR168a	+ + +	High	CAT	+ +	Moderate
APX	+ + +	High	miR399g	+ +	Moderate
PA	+ +	Moderate	SOD	+ +	Moderate
MDA	+ +	Moderate	miR828a	+ +	Moderate
GPOX	+ +	Moderate	RT	+ +	Moderate
SOD	+ +	Moderate	miR168a	+ +	Moderate
DPPH	+ +	Moderate	PA	+	Weak
RL	+ +	Moderate	APX	+	Weak
PP	-	Weak	PP	+	Weak
LRWC	-	Weak	RDW	+	Weak
RD	-	Weak	RFW	-	Weak
RT	–	Moderate	LRWC	–	Moderate
RF	–	Moderate	RV	–	Moderate
LN	––	High	SL	–	Moderate
RV	––	High	GPOX	–	Moderate
RFW	––	High	SDW	–	Moderate
RDW	––	High	LN	–	Moderate
SFW	––	High	SFW	–	Moderate
SL	––	High	RD	––	High
SDW	––	High	RF	––	High

Only independent variables were analysed. Pearson’s correlation coefficients are represented in Table S3. Correlation markings and annotations: *r* = 1, ‛ +  +  +  + ’, perfect; |*r*|≥ 0.7, ‛ +  +  + ’ or ‛–-’, high; 0.7 >|*r*|> 0.3, ‛ +  + ’ or ‛–’, moderate; |*r*|≤ 0.3, ‛ + ’ or ‛-’, weak; *r* = 0, ‛––’, unrelated (Ratner 2009). *APX* ascorbate peroxidase, *CAT* catalase, *D* diameter, *DPPH* 2,2-diphenyl-1-picrylhydrazyl, *DW* dry weight, *F* fork, *FW* fresh weight, *GPOX* guaiacol peroxidase, *L* length, *LN* leaf number, *LRWC* leaf relative water content, *MDA* malondialdehyde, *PA* phenolic acids, *PP* polyphenols, *R* root, *S* shoot, *SOD* superoxide dismutase, *T* tip, *V* volume

**Fig. 12:**
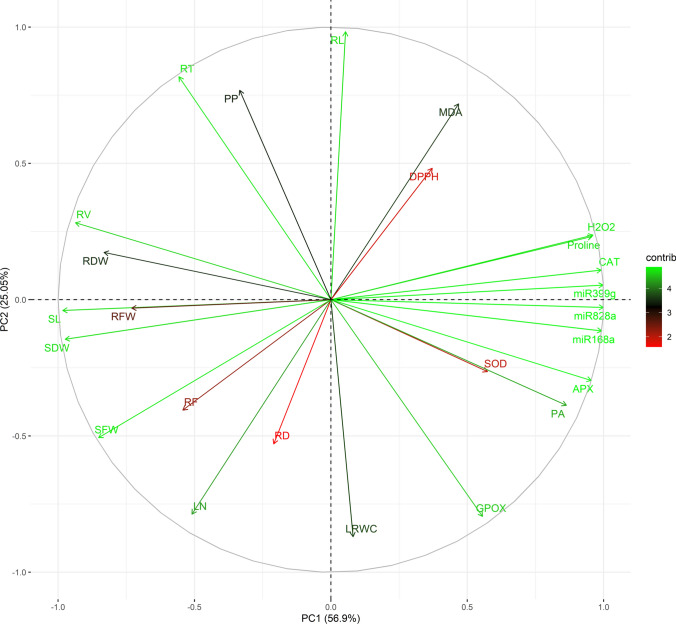
Principal component analysis (PCA) using the tested independent variables. Data were obtained after stressing two flax genotypes, Flanders and Astella, with 100 mM NaCl for 1 week. Data of 26 independent variables were logarithmically transformed, standardised, and normalised for this analysis. The PCA plot represents the loading values of different variables measured in this study. The PC1 and PC2 describe a total of 81.95% variation. *APX* ascorbate peroxidase, *CAT* catalase, *D* diameter *DPPH* 2,2-diphenyl-1-picrylhydrazyl, *DW* dry weight, *F* fork, *FW* fresh weight, *GPOX* guaiacol peroxidase, *L* length, *LN* leaf number, *LRWC* leaf relative water content, *MDA* malondialdehyde, *PA* phenolic acids, *PP* polyphenols, *R* root, *S* shoot, *SOD* superoxide dismutase, *T* tip, *V* volume

The findings of PCC were verified using PCA, and the most important variables of this study were identified. A cluster of H_2_O_2_, proline, CAT, miR168a, miR399g, and miR828a was found in the PCA plot, thereby confirming their positive correlation with salinity stress. Several morphology-related variables (RV, SL, SDW, RDW, and RFW) were positioned almost opposite to this identified cluster, showing their inverse relationship with salt stress. The orthogonal positions of RL, PP, RD, and LRWC relative to the cluster suggested their minimal contribution to salinity stress (Fig. 12). Principal components (PCs) with an eigenvalue > 1 were considered for further analysis. The first two PCs, PC1 and PC2, cumulatively accounted for 81.95% of the observed variance of this study. The PC1 (56.90% variance) explained the most significantly contributing variables of this study. The order of loading values explained the magnitude of the contributions of the variables to their respective principal component. The loading value of H_2_O_2_ (|*r*|= 0.25) was higher than that of MDA (|*r*|= 0.12) in PC1. The higher loading values of miR828a, miR399g, miR168a, CAT, SL, and SDW than H_2_O_2_ confirmed their strong correlation with the salinity markers, and were designated as the most contributing salinity stress variables. PC2 (25.05% variance) explained the less important study variables. The loading of PP in PC2 over H_2_O_2_ and MDA confirmed its minimal contribution in this study (Table 3).

**Table 3:** Variable loadings, eigenvalues, and variances in principal component analysis

Variables	PC1	Variables	PC2	Variables	PC3
*Positive loadings*					
***miR828a***	***0.2648978***	***RL***	***0.39289522***	***RD***	***0.38710509***
***miR399g***	***0.26461649***	***RT***	***0.32681686***	***RF***	***0.34633794***
***miR168a***	***0.2632747***	***PP***	***0.30693646***	***PP***	***0.25714085***
***CAT***	***0.26264676***	***MDA***	***0.28709765***	**GPOX**	**0.11428969**
***H***_***2***_***O***_***2***_	***0.25476642***	**DPPH**	**0.19263552**	**RV**	**0.09818402**
*Proline*	*0.25379563*	**RV**	**0.11276203**	**RL**	**0.0823279**
*APX*	*0.25308142*	**H**_**2**_**O**_**2**_	**0.09473045**	**Proline**	**0.08146993**
*PA*	*0.22893509*	Proline	0.0926391	**H**_**2**_**O**_**2**_	**0.0673606**
*SOD*	*0.15217154*	RDW	0.06933241	CAT	0.03903071
*GPOX*	*0.14739423*	CAT	0.04338672	miR399g	0.01501916
*MDA*	*0.12411768*	miR399g	0.02140551	miR828a	0.0144558
DPPH	0.09848947			miR168a	0.01376251
LRWC	0.02150049				
RL	0.01422691				
*Negative loadings*					
***SL***	***− 0.2605821***	***LRWC***	***− 0.3475668***	***DPPH***	***-0.37352668***
***SDW***	***− 0.2582573***	***GPOX***	***− 0.3177062***	***SOD***	***-0.36490261***
*RV*	*− 0.2482749*	***LN***	***− 0.3144554***	***RFW***	***-0.32128967***
*SFW*	*− 0.2258609*	**RD**	**− 0.21142635**	***RDW***	***-0.24823586***
*RDW*	*− 0.2205236*	**SFW**	**− 0.20237608**	***MDA***	***-0.24221286***
*RFW*	*− 0.1935944*	**RF**	**− 0.16183992**	**LRWC**	**-0.22918363**
*RT*	*− 0.1476995*	**PA**	**− 0.15492268**	**LN**	**-0.16389061**
*RF*	*− 0.1439234*	**APX**	**− 0.11854756**	**PA**	**-0.15190213**
*LN*	*− 0.1351009*	**SOD**	**− 0.10548105**	**SL**	**-0.08482107**
PP	− 0.08861936	SDW	− 0.0581337	**SDW**	**-0.08157007**
RD	− 0.0555144	miR168a	− 0.04575571	**RT**	**-0.06805448**
		SL	− 0.01578449	SFW	-0.0629139
		RFW	− 0.01247515	APX	-0.01368798
		miR828a	− 0.01147761		
*PCA result*					
Eigenvalue	14.23		6.26		4.51
Variance	56.90		25.05		18.05

Only independent variables were analysed. Principal components (PCs) with eigenvalue > 1 were considered. |Values| ≥|loadings| of H_2_O_2_ and MDA were marked in bold and italics, respectively. *APX* ascorbate peroxidase, *CAT* catalase, *D* diameter, *DPPH* 2,2-diphenyl-1-picrylhydrazyl, *DW* dry weight, *F* fork, *FW* fresh weight, *GPOX* guaiacol peroxidase, *L* length, *LN* leaf number, *LRWC* leaf relative water content, *MDA* malondialdehyde, *PA* phenolic acids, *PP* polyphenols, *R* root, *S* shoot, *SOD* superoxide dismutase, *T* tip, *V* volume

## Discussion

Several abiotic stresses, including salinity stress, reduce the productivity of the lignan-rich multi-utilitarian crop flax (Goyal et al. 2014; Yadav et al. 2024). Flax salt tolerance has been assessed in many studies (Li et al. 2022a, b; Wang et al. 2022; Yadav et al. 2024). Lignans have been reported to mitigate abiotic stress in plants other than flax (Ghotbzadeh Kermani et al. 2019; Sharma et al. 2019; Mwamba et al. 2020). Consequently, the present study investigated the previously unexplored role of lignans in flax salt stress mitigation for the first time. Two flax genotypes, Flanders and Astella, with contrasting lignan content (SDG: 7.75 mg/g and 13.07 mg/g, respectively) were selected for this study, and their performance under salinity stress was compared. The application of 100 mM NaCl stress was conducted in accordance with the methodologies described in previous reports on salinity stress studies in flax (Hussein and Alshammari 2022; Li et al. 2022a; Wang et al. 2022). The present experiments were conducted 21 days after the germination of the flax plants, as flax undergoes a transition from slower to faster growth at this stage (Li et al. 2022b). Salinity initiates two types of stress in plants: osmotic and ionic. Osmotic stress primarily affects the morphology of plants. In ionic stress, an imbalance in physiological ion and nutrient homeostasis leads to oxidative stress-mediated cell damage (Munns and Tester 2008). In this study, various morphological and biochemical markers were analysed to understand the effect of these two phases. In addition, genomic and microscopic assays were performed to facilitate a comprehensive understanding of the study.

### Salinity stress caused more growth arrest in Flanders than in Astella

Morphological analyses revealed that salt stress decreased multiple morphological parameters (SL, RD, RV, RT, RF, LN, SFW, SDW, RFW, RDW, and LRWC) in both Flanders and Astella, suggesting growth impairment under stress (Figs. 1, 2, 3, 4; Table S2). Under saline conditions, reduced soil water and nutrient availability lead to physiological drought in plants, initiating osmotic stress. Plants respond to the stress immediately by reprogramming gene expression and altering hormonal homeostasis, which decreases cell division, cell elongation, and meristem size and activity, thus affecting growth (Bartels and Sunkar 2005; Munns and Tester 2008). Salinity-mediated growth arrest has frequently been observed in various plants (Zeeshan et al. 2020; Lu et al. 2023), including flax (Li et al. 2022a, b; Wang et al. 2022).

The morphology of the tested genotypes was affected differently. Among the salt-stressed genotypes, most of the root parameters of Flanders were decreased (RL, RD, RV, and RT), indicating its more root growth arrest than Astella (Figs. 1, 2; Table S2). Such behaviour of Flanders suggested the reduction of the root’s contact with the saline soil to minimise salt uptake and reflected a better adaptive strategy against salinity (Yu et al. 2022). Under salinity, certain parameters were increased (LN, SFW, and LRWC) in Flanders over Astella, and the rest remained unchanged (Figs. 1, 2, 3, 4; Table S2). Such non-uniform observations have been previously reported and explain the genotype-specificity of stress tolerance (Robin et al. 2016; Saddiq et al. 2021).

Salt stress affected the shoots and roots of Flanders and Astella differently. Under saline conditions, the > 1 ratio of RFW/SFW and RDW/SDW represented increased biomass of roots over shoots in both salt-treated flax genotypes (Table 1). Under stress, the higher RDW/SDW ratio evidenced greater root biomass of Flanders than Astella. Coupled with the arrested root growth, such high root biomass drastically increased the root mass density (RMD) of Flanders over Astella (Fig. 4; Table S2). These changes could have happened due to improved physiological adaptations in Flanders, such as improved morphology or enhanced ion exclusion mechanisms to reduce physiological salt concentration (Bartels and Sunkar 2005; Acosta-Motos et al. 2017).

Relative water contents are important plant traits for assessing salinity stress. The higher LRWC of salt-treated Flanders than Astella (Fig. 4b; Table S2) and their unchanged SRWC and RRWC across all treatments (Fig. 3b; Table S2) signified that, despite the similar water content of shoots and roots, the leaves of Flanders stored more water under salt stress than those of Astella. A higher LRWC often represents better salinity stress management in various plants (Esringü et al. 2011; Boussora et al. 2024), including flax (Li et al. 2022b; Siddiqui et al. 2022).

The results showed that under salinity, Flanders restricted its shoot and root growth more and retained more leaf water than Astella. In practice, Flanders was arresting its growth and saving vital resources under stress, a well-known stress-adaptive phenomenon.

### Salinity stress harmed Astella more than Flanders

Plant cells maintain a dynamic balance between naturally occurring ROS molecules, such as hydrogen peroxide (H_2_O_2_), and ROS-scavenging antioxidants to achieve a neutral redox potential state (Hasanuzzaman et al. 2019). An overwhelming salinity stress alters this cellular neutral redox state by generating excessive ROS molecules, which destroy the cell membrane by lipid peroxidation and lead to death (Gill and Tuteja 2010). Lipid peroxidation generates MDA. The H_2_O_2_ and MDA are considered important stress markers (Wang et al. 2022). Elevated ROS and lipid peroxidation are associated with salt susceptibility (Kumar et al. 2018; Zeeshan et al. 2020). In this study, the elevated stress markers in salt-treated Flanders and Astella (Fig. 5; Table S3) suggested their cellular oxidative stress conditions under salinity. In addition, the significantly higher stress markers in Astella represented greater ROS-mediated cell damage and lower salt tolerance than in Flanders, indicating a more efficient ROS-scavenging system in Flanders.

### Salinity stress hyperactivated the antioxidant system in Flanders

Plants activate the antioxidant defence system to mitigate oxidative stress (Gill and Tuteja 2010; Hasanuzzaman et al. 2019). In this study, enzymatic antioxidants (SOD, CAT, APX, and GPOX) were significantly induced in Flanders than in Astella under stress compared to their respective controls (Fig. 6a–d; Table S3). The SOD provides frontline defence against ROS by dismuting O_2_^•–^ to H_2_O_2_ (Gill and Tuteja 2010). The H_2_O_2_ is quickly scavenged by CAT, GPOX, and APX and converted to H_2_O and O_2_ (Hasanuzzaman et al. 2019; Mekawy et al. 2020). In plants, H_2_O_2_ has a dual role: (1) at low concentrations, it acts as a signalling molecule to activate various pathways that alleviate stress; (2) at high concentrations, it acts as a toxic substance and initiates programmed cell death (Saxena et al. 2016). In this study, the lower concentration of H_2_O_2_ must have hyperactivated the antioxidant system in salinity-stressed Flanders compared to Astella under stress.

Non-enzymatic antioxidants such as PP and its subcategory PA are powerful ROS scavengers in plants, and serve as important stress markers (Šamec et al. 2021). In this study, PP and PA were upregulated in Flanders but downregulated in Astella under salt stress (Fig. 7a, b; Table S3). Salt stress can alter PP levels by affecting key enzymes in the phenylpropanoid biosynthesis pathway (Sharma et al. 2019). While PP and PA levels generally increase under salinity (Ozfidan-Konakci et al. 2015; Sarker et al. 2023), contrary findings also exist (Mahmoudi et al. 2010; Slama et al. 2017). Findings of this study suggested that ROS-mediated photosynthetic apparatus damage, photosynthesis reduction (low LN), and antioxidant biosynthetic enzyme impairments may have lowered non-enzymatic antioxidants in Astella; the lower radical-scavenging activity in Astella also supports this fact (Fig. 7c; Table S3).

Higher antioxidant activity suggests improved salinity tolerance (Kumar et al. 2018). In this study, a significant rise in the radical-scavenging potential of Flanders over Astella was observed under stress, indicating a more robust antioxidant system and greater salinity resilience in Flanders.

### Proline was providing multi-dynamic protection to Flanders

Proline is a crucial salinity stress index with multifaceted stress ameliorative roles (Mansour and Ali 2017). Salt treatment-mediated proline upregulation in tolerant genotypes has already been reported in different plants (Kumar et al. 2018; Nguyen et al. 2021), including flax (Li et al. 2022b). In this study, the significantly higher proline accumulation in Flanders under salt stress suggested that this genotype can withstand salt stress better than Astella (Fig. 7d; Table S3). Moreover, lower proline accumulation in stressed Astella signified reduced protection against salinity, which could also explain its elevated ROS level (Fig. 5a) and decreased antioxidant activity (Figs. 6a–d, 7a–c) under salt stress.

### Lignan-related miRNAs were upregulated under salt stress

The expression levels of three lignan biosynthesis-related miRNAs: miR168a, miR399g, and miR828a, were analysed using the two-tailed qPCR (TT-qPCR) assay. The TT-qPCR assay was originally designed by Androvic et al. (2017) and, to date, very few reports of its use to measure plant miRNA expression exist (Barczak-Brzyżek et al. 2019, 2022; Kong et al. 2022; Pawełkowicz et al. 2022). This study reports a kit-based TT-qPCR assay for the first time. The expressions of the tested miRNAs were upregulated in both genotypes under salt stress (Fig. 8).

The miR168 (Xia et al. 2023), miR399 (Baek et al. 2016), and miR828 (Shuai et al. 2016) family members are known to be expressed under saline conditions in different plants, including flax (Barvkar et al. 2013; Yu et al. 2016). These miRNAs regulate the phenylpropanoid pathway indirectly via certain mediators. The miR168 alters the expression of CYP450, a critical controller of lignan biosynthesis (Barvkar et al. 2013; Zhang et al. 2022). The miR399 regulates the phenylalanine ammonia lyase (PAL) gene of the phenylpropanoid pathway. The PAL performs the first and committed step of the phenylpropanoid pathway (Yan et al. 2019). The miR828 upregulates various MYB transcription factors (TFs) (Deng and Lu 2017), which are important regulators of the phenylpropanoid pathway (Markulin et al. 2019a, b; Ražná et al. 2022).

In addition, miR168 downregulates AGO1 of RISC via feedback inhibition (Ding et al. 2009). Such interference with the core miRNA processing hub results in the global readjustment of cellular metabolic processes under salt stress (Carnavale Bottino et al. 2013). Islam et al. (2022) proposed that miR399 is involved in the ABA metabolic pathway during salt stress.

These results depicted that, unlike most miRNAs, the tested miR168a, miR399g, and miR828a regulated multiple target sequences across numerous biological processes. It is a densely interconnected regulatory network, and its pleiotropic effect is evident (Wang et al. 2024). Different secondary metabolites of plants, including lignans, are biosynthesised via the phenylpropanoid pathway. Therefore, this study provided an indication, rather than direct evidence, for the involvement of lignans in salt stress mitigation in flax.

### Salt stress affected the viability of root cells

Differential staining of flax roots showed the viable cells in the root tips and lateral root buds as green by FDA staining, while damaged or dead root epidermal cells appeared red after PI staining (Fig. 9a–h). Plant roots are the primary organs affected by soil salinity (AbdElgawad et al. 2016). Since high salt concentrations in a soil induce physiological drought in plants, they respond similarly to drought and salt stress. Duan et al. (2010) previously reported that salt stress-mediated ROS formation damaged the root cells, especially in the meristematic and elongation zones, and ceased root growth. In this study, such root damage due to NaCl stress may have arrested root growth quickly in Flanders than in Astella (Figs. 1, 2). In addition, direct exposure of root epidermis cells (the outermost layer of roots) to high soil salinity triggered an extensive oxidative burst, causing the maximum damage to these cells.

### Multivariate analysis correlated study variables and coined the most vital salt stress-responsive variables

The abundance of measured variables in treatment samples was evaluated using heatmap analysis. Then, the tested variables were correlated through PCC to understand their cumulative effect on salt stress in the two studied flax genotypes. Next, PCA revealed the most important variables of the study.

Heatmap analysis showed that the abundance pattern was substantially different between the salt-treated samples (Fig. 10). This phenomenon suggested a genotype-dependent differential regulation of the salt stress response, corroborated the earlier findings of this study, and has been well documented previously (Nefissi Ouertani et al. 2022; Khan et al. 2023).

The salt stress markers H_2_O_2_ and MDA represent the extent of salinity-derived ROS generation and associated cell damage, respectively. Therefore, the correlation between these salt stress markers and the tested variables was determined using PCC (Fig. 11; Table 2; S4). In this study, the high negative correlations of most of the tested morphological trait variables (LN, RV, RFW, RDW, SFW, SL, SDW, RD, and RF) with the salt stress markers H_2_O_2_ and MDA suggested an inverse relationship between salinity and plant growth. It corroborated the earlier findings of this study on salt stress-mediated growth arrest (Figs. 1, 2, 3, 4) by elevated ROS and cell damage. Such findings have also been reported in other studies (Mekawy et al. 2020; Li et al. 2022a, b; Wang et al. 2022; Lu et al. 2023). The salt stress markers H_2_O_2_ and MDA had a highly positive correlation with antioxidants (CAT, APX, and DPPH), proline, and miRNAs, suggesting their simultaneous activation under salt stress. The co-upregulation and salinity-ameliorate role of antioxidants and proline to minimise ROS has been previously established (Hasanuzzaman et al. 2019; Li et al. 2022b) and is also reflected in this study (Figs. 6a–d, 7a–d). The strong positive correlations between miRNAs and ROS in this study explained the salinity-mediated upregulation of these miRNAs (Fig. 8a–c), supporting the connection of these miRNAs with salt stress mitigation. However, the precise mechanism remains to be elucidated. The correlations of PP with the salt stress markers H_2_O_2_ and MDA were weak. Since lignans are a type of PP, findings of PCC could not provide substantial evidence to establish the link between lignans and the salinity stress response.

The PCA confirmed the findings of PCC analysis and established miR828a, miR399g, miR168a, CAT, SL, and SDW as the most influential salt stress-responsive variables of this investigation (Fig. 12; Table 3). Moreover, PCA also showed that PP contributed negligibly to the study. Throughout this study, the salinity stress adaptiveness of the high lignan-containing genotype Astella was weaker than that of the low lignan-containing genotype Flanders. These results suggested that despite being biosynthesised by the studied miRNAs, lignans are not crucial for salinity stress adaptiveness. Therefore, it was hypothesised that in addition to lignan biosynthesis, these miRNAs could have more elaborate roles in the salinity response. Another factor(s) or content component(s) biosynthesised by these miRNAs could be involved in the increased salinity stress-adaptive capacity of Flanders. In addition to the miRNAs, the enzymatic antioxidant CAT also influenced the outcomes of this study. The CAT is known for its oxidative stress mitigation property under stress (Hasanuzzaman et al. 2019; Mekawy et al. 2020). The remaining two influential variables, SL and SDW, could be considered morphological markers of salt stress adaptation in flax.

## Conclusion

This paper reports the salt stress-adaptive differential responses of two flax genotypes, Flanders and Astella, having contrasting lignan contents. Under NaCl salt stress, arrested growth, lower ROS generation, lower cell damage, higher activities of antioxidant system components, higher amount of proline, and upregulated lignan biosynthesis-related miRNAs were observed in Flanders over Astella. This study presents the first report on a kit-based two-tailed qPCR (TT-qPCR) assay. Root viability assay using differential staining depicted the existence of viable root tips despite ROS-mediated cell damage. Multivariate analysis revealed the relationships between the tested variables and established miR828a, miR399g, miR168a, CAT, SL, and SDW as the most influential salt stress-responsive variables. However, no correlation was found between lignans and salt stress adaptiveness. It was hypothesised that some undiscovered factors, besides the most influential variables, were also responsible for the improved salinity adaptiveness.

## Supplementary Information

Below is the link to the electronic supplementary material.

Supplementary file1 (PDF 249 KB)

## Data Availability

The data supporting the findings of this study are available from the corresponding author upon reasonable request.

## Abbreviations

APX: Ascorbate peroxidase
CAT: Catalase
DPPH: 2,2-Diphenyl-1-picrylhydrazyl
FDA: Fluorescein diacetate
GPOX: Guaiacol peroxidase
LN: Leaf number
MDA: Malondialdehyde
PA: Phenolic acids
PCC: Pearson’s correlation coefficient
PI: Propidium iodide
PP: Polyphenols
R: Root
RWC: Relative water content
S: Shoot
SOD: Superoxide dismutase
