# Supplementary material for: A comparative analysis of the adaptability of salt stress between two flax (Linum usitatissimum L.) genotypes, Flanders and Astella, having contrasting lignan contents
Source: Planta. 2025 Nov 10;262(6):150. doi: 10.1007/s00425-025-04861-4 (PMC12602683; doi:10.1007/s00425-025-04861-4)
Supplement: Supplementary file 1 — Supplementary file1 (PDF 249 KB) [file 425_2025_4861_MOESM1_ESM.pdf]

SUPPLEMENTARY TABLES FOR THE ORIGINAL ARTICLE TITLED

A comparative analysis of the adaptability of salt stress between two flax (*Linum usitatissimum* L.) genotypes, Flanders and Astella, having contrasting lignan contents

RUNNING TITLE

Lignan-mediated salt stress adaptiveness in flax

ARTICLE TYPE

Original Article

AUTHORS

Anirban Jyoti Debnath[1] (Corresponding author) (anirbandebnath@ymail.com) (ORCID ID: 0000-0003-1907-837X)

Ľubomír Harenčár[3] (harencar.l@gmail.com) (ORCID ID: 0000-0002-2372-6539)

Matúš Kučka[4] (matus.kucka@upjs.sk) (ORCID ID: 0000-0003-1219-3519)

Marek Kovár[1] (marek.kovar@uniag.sk) (ORCID ID: 0000-0002-1478-8383)

Eva Ivanišová[2] (eva.ivanisova@uniag.sk) (ORCID ID: 0000-0001-5193-2957)

Veronika Mistríková[3] (veronika.mistrikova@savba.sk) (ORCID ID: 0000-0003-4268-6189)

Ján Gažo[1] (jan.gazo@uniag.sk) (ORCID ID: 0000-0002-8528-9223)

Katarína Ražná[1] (Corresponding author) (katarina.razna@uniag.sk) (ORCID ID: 0000-0003-2121-131X)

ADDRESSES OF AUTHORS

[1] Institute of Plant and Environmental Sciences,

Faculty of Agrobiology and Food Resources,

Slovak University of Agriculture in Nitra,

Trieda Andreja Hlinku 2,

949 76 Nitra, Slovakia

[2] Institute of Food Sciences,

Faculty of Biotechnology and Food Sciences,

Slovak University of Agriculture in Nitra,
Trieda Andreja Hlinku 2,
949 76 Nitra, Slovakia

[3] Institute of Plant Genetics and Biotechnology,
Plant Science and Biodiversity Centre,
Slovak Academy of Sciences,
P.O.Box 39 A, Akademická 2,
950 07 Nitra, Slovakia

[4] Department of Genetics,
Institute of Biology and Ecology,
Faculty of Science,
Pavol Jozef Šafárik University,
Mánesova 23,
041 54 Košice, Slovakia

Supplementary Table S1 Primers used in this study. EIF5A, eukaryotic transcription initiation factor 5A

Gene	Forward Primer	Reverse Primer
Cyclophilin	5'-TGATTGCGGTCAGCTGTAAG-3'	5'-AGGTGAAACGCTAGGCAGAA-3'
ETIF5A	5'-TGCCACATGTGAACCGTACT-3'	5'-CTTTACCCTCAGCAAATCCG-3'

Supplementary Table S2 Summary of various morphological parameter changes of two flax genotypes, Flanders and Astella, under 100 mM NaCl treatment. The data are relative changes under the stress period, normalised against control treatments, and represented as percentage (%) change from the control treatments. The positive (+) and negative (-) markings indicate an increase and decrease in values from the respective controls, respectively. Control treatment values are omitted.

**Morphological parameters**	**Percentage change**	
	**Flanders**	**Astella**
Shoot length (SL)	-20.79	-18.87
Root length (RL)	-20.12	+71.81
Root diameter (RD)	-6.76	-156.94
Root volume (RV)	-81.06	-29.55
Root tip number (RT)	-73.30	+42.80
Root fork number (RF)	-27.06	-7.5
Shoot fresh weight (SFW)	-38.94	-57.30
Root fresh weight (RFW)	-17.67	-42.48
Shoot dry weight (SDW)	-53.61	-54.75
Root dry weight (RDW)	-46.34	-53.85
Shoot relative water content (SRWC)	+2.93	-0.65
Root relative water content (RRWC)	+3.47	+2.38
Leaf relative water content (LRWC)	+5.23	-7.07
Leaf number (LN)	-1.90	-45.31

Supplementary Table S3 Summary of different biochemical parameter changes of two flax genotypes, Flanders and Astella, under 100 mM NaCl treatment. The data are represented as percentage (%) change from the respective control treatments. The positive (+) and negative (-) markings indicate an increase and decrease in values from the respective controls, respectively

**Biochemical parameters**	**Percentage change**	
	**Flanders**	**Astella**
Hydrogen peroxide (H_2O_2)	+256.17	+661.71
Malondialdehyde (MDA)	+270.73	+457.03
Superoxide dismutase (SOD)	+178.29	+121.83
Catalase (CAT)	+1178.78	+609.27
Ascorbate peroxidase (APX)	+183.11	+139.74
Guaiacol peroxidase (GPOX)	+352.54	+224.20
Polyphenols (PP)	+35.70	-37.10
Phenolic acids (PA)	+9.41	-48.62
2,2-Diphenyl-1-picrylhydrazyl (DPPH)	+32.36	-41.90
Proline	+1175.61	+857.61

Supplementary Table S4 Pearson's correlation coefficients between the tested independent variables. APX, ascorbate peroxidase; CAT, catalase; D, diameter; DPPH, 2,2-diphenyl-1-picrylhydrazyl; DW, dry weight; F, fork; FW, fresh weight; GPOX, guaiacol peroxidase; L, length; LN, leaf number; LRWC, leaf relative water content; MDA, malondialdehyde; PA, phenolic acids; PP, polyphenols; R, root; S, shoot; SOD, superoxide dismutase; T, tip; V, volume

	SL	RL	RD	RV	RT	RF	SFW	SDW	RFW	RDW	LRWC	LN	SOD
SL	1	-0.12308	0.078503	0.871619	0.541269	0.416938	0.881344	0.994323	0.841871	0.905647	0.042379	0.59463	-0.41399
RL	-0.12308	1	-0.38752	0.263629	0.7488	-0.29858	-0.56694	-0.22559	-0.18924	0.033697	-0.93582	-0.8618	-0.36427
RD	0.078503	-0.38752	1	0.218324	-0.43489	0.932957	0.336364	0.138409	-0.39189	-0.3513	0.042745	0.236652	-0.61802
RV	0.871619	0.263629	0.218324	1	0.722247	0.547521	0.626955	0.834949	0.532582	0.717823	-0.42288	0.182525	-0.77362
RT	0.541269	0.7488	-0.43489	0.722248	1	-0.13514	0.079766	0.448727	0.479917	0.681464	-0.68601	-0.30927	-0.42351
RF	0.416938	-0.29858	0.932957	0.547521	-0.13514	1	0.569168	0.460173	-0.09317	-0.00675	-0.05004	0.339089	-0.77502
SFW	0.881344	-0.56694	0.336364	0.626955	0.079766	0.569168	1	0.926614	0.729057	0.691179	0.436403	0.879074	-0.25167
SDW	0.994323	-0.22559	0.138409	0.834949	0.448727	0.460173	0.926614	1	0.834062	0.876327	0.131889	0.671139	-0.38634
RFW	0.841871	-0.18924	-0.39189	0.532582	0.479917	-0.09317	0.729057	0.834062	1	0.961848	0.300246	0.634271	0.118255
RDW	0.905647	0.033697	-0.3513	0.717823	0.681464	-0.00675	0.691179	0.876327	0.961848	1	0.03842	0.470923	-0.11437
LRWC	0.042379	-0.93582	0.042745	-0.42288	-0.68601	-0.05004	0.436403	0.131889	0.300246	0.03842	1	0.812512	0.653512
LN	0.59463	-0.8618	0.236652	0.182525	-0.30927	0.339089	0.879074	0.671139	0.634271	0.470923	0.812512	1	0.185116
SOD	-0.41399	-0.36427	-0.61802	-0.77362	-0.42351	-0.77502	-0.25167	-0.38634	0.118255	-0.11437	0.653512	0.185116	1
CAT	-0.99285	0.174391	-0.19667	-0.87971	-0.47506	-0.52071	-0.90995	-0.9951	-0.78332	-0.84884	-0.05446	-0.61908	0.475635
APX	-0.92121	-0.24549	-0.06685	-0.98362	-0.77014	-0.41943	-0.65906	-0.88161	-0.66789	-0.83007	0.34955	-0.24287	0.648694
GPOX	-0.55874	-0.70927	0.503841	-0.69425	-0.99492	0.198799	-0.10346	-0.46794	-0.54683	-0.72835	0.618268	0.257718	0.340688
H2O2	-0.97956	0.309629	-0.20892	-0.80308	-0.36214	-0.51232	-0.95774	-0.99526	-0.8067	-0.83357	-0.1979	-0.72597	0.378012
MDA	-0.39576	0.641395	-0.90123	-0.343	0.401088	-0.92363	-0.69381	-0.47133	-0.01305	0.006608	-0.3363	-0.62466	0.477948
Proline	-0.98116	0.30953	-0.18074	-0.79487	-0.36871	-0.48616	-0.95597	-0.99613	-0.82432	-0.84724	-0.20824	-0.73043	0.354057
DPPH	-0.24115	0.35504	-0.98534	-0.37736	0.301969	-0.98068	-0.45449	-0.29445	0.255316	0.193109	-0.00275	-0.29229	0.701104
PP	0.199742	0.832675	0.112889	0.643642	0.735307	0.27233	-0.1772	0.119197	-0.15276	0.123182	-0.96103	-0.6242	-0.81801
PA	-0.7752	-0.39122	-0.24108	-0.98526	-0.75139	-0.54916	-0.49614	-0.72878	-0.39814	-0.61527	0.564279	-0.02261	0.848058
miR168a	-0.9767	-0.05416	-0.1233	-0.95607	-0.65103	-0.47116	-0.79184	-0.95566	-0.74145	-0.8612	0.165865	-0.42608	0.57748
miR399g	-0.98879	0.111794	-0.21107	-0.91283	-0.51681	-0.54	-0.88161	-0.9855	-0.75221	-0.83766	0.018818	-0.56182	0.533959
miR828a	-0.98637	0.030748	-0.16875	-0.93729	-0.58451	-0.50813	-0.84069	-0.97435	-0.7496	-0.85219	0.091048	-0.4972	0.557213

	CAT	APX	GPOX	H2O2	MDA	Proline	DPPH	PP	PA	miR168a	miR399g	miR828a
SL	-0.99285	-0.92121	-0.55874	-0.97956	-0.39576	-0.98116	-0.24115	0.199742	-0.7752	-0.9767	-0.98879	-0.98637
RL	0.174391	-0.24549	-0.70927	0.309629	0.641395	0.30953	0.35504	0.832675	-0.39122	-0.05416	0.111794	0.030748
RD	-0.19667	-0.06685	0.503841	-0.20892	-0.90123	-0.18074	-0.98534	0.112889	-0.24108	-0.1233	-0.21107	-0.16875
RV	-0.87971	-0.98362	-0.69425	-0.80308	-0.34299	-0.79487	-0.37736	0.643642	-0.98526	-0.95607	-0.91283	-0.93729
RT	-0.47506	-0.77014	-0.99492	-0.36214	0.401088	-0.36871	0.301969	0.735307	-0.75139	-0.65103	-0.51681	-0.58451
RF	-0.52071	-0.41943	0.198799	-0.51232	-0.92363	-0.48616	-0.98068	0.27233	-0.54916	-0.47116	-0.54	-0.50813
SFW	-0.90995	-0.65906	-0.10346	-0.95774	-0.69381	-0.95597	-0.45449	-0.1772	-0.49614	-0.79184	-0.88161	-0.84069
SDW	-0.9951	-0.88161	-0.46794	-0.99526	-0.47133	-0.99613	-0.29445	0.119197	-0.72878	-0.95566	-0.9855	-0.97435
RFW	-0.78332	-0.66789	-0.54683	-0.8067	-0.01305	-0.82432	0.255316	-0.15276	-0.39814	-0.74145	-0.75221	-0.7496
RDW	-0.84884	-0.83007	-0.72835	-0.83357	0.006608	-0.84724	0.193109	0.123182	-0.61527	-0.8612	-0.83766	-0.85219
LRWC	-0.05446	0.34955	0.618268	-0.1979	-0.3363	-0.20824	-0.00275	-0.96103	0.564279	0.165865	0.018818	0.091048
LN	-0.61908	-0.24287	0.257718	-0.72597	-0.62466	-0.73043	-0.29229	-0.6242	-0.02261	-0.42608	-0.56182	-0.4972
SOD	0.475635	0.648694	0.340688	0.378012	0.477948	0.354057	0.701104	-0.81801	0.848058	0.57748	0.533958	0.557213
CAT	1	0.910984	0.48454	0.989494	0.499074	0.987784	0.354526	-0.20244	0.786538	0.973679	0.997159	0.989178
APX	0.910984	1	0.759419	0.842752	0.248719	0.839938	0.234677	-0.56276	0.948604	0.980972	0.935875	0.96133
GPOX	0.48454	0.759419	1	0.380494	-0.43579	0.389896	-0.36935	-0.66376	0.709859	0.65015	0.520012	0.585723
H2O2	0.989494	0.842752	0.380494	1	0.546487	0.99953	0.357657	-0.06095	0.691648	0.931215	0.976296	0.95764
MDA	0.499074	0.248719	-0.43579	0.546487	1	0.525591	0.928484	0.114195	0.291779	0.367561	0.489285	0.431287
Proline	0.987784	0.839938	0.389896	0.99953	0.525591	1	0.32999	-0.04738	0.680843	0.929055	0.973318	0.955053
DPPH	0.354526	0.234677	-0.36935	0.357657	0.928484	0.32999	1	-0.18742	0.389963	0.290483	0.37125	0.332932
PP	-0.20244	-0.56276	-0.66376	-0.06095	0.114195	-0.04738	-0.18742	1	-0.76262	-0.40387	-0.27503	-0.33923
PA	0.786538	0.948604	0.709859	0.691648	0.291779	0.680843	0.389963	-0.76262	1	0.892374	0.830736	0.863935
miR168a	0.973679	0.980972	0.65015	0.931215	0.367561	0.929055	0.290483	-0.40387	0.892374	1	0.985862	0.996302
miR399g	0.997159	0.935875	0.520012	0.976296	0.489285	0.973318	0.37125	-0.27503	0.830736	0.985862	1	0.996614
miR828a	0.989178	0.96133	0.585723	0.95764	0.431287	0.955053	0.332932	-0.33923	0.863935	0.996302	0.996614	1
